# Dipoles affect conformational equilibrium^☆^

**DOI:** 10.1016/j.jphotochem.2025.116362

**Published:** 2025-03-02

**Authors:** Eli M. Espinoza, J. Omar O’Mari, James B. Derr, Mimi Karen Billones, John A. Clark, Maryann Morales, Tomasz Szreder, Bing Xia, Javier Ceballos, Ctirad Červinka, Valentine I. Vullev

**Affiliations:** a Department of Chemistry, University of California, Riverside, CA 92521, USA; b Department of Bioengineering, University of California, Riverside, CA 92521, USA; c Department of Biochemistry, University of California, Riverside, CA 92521, USA; d Institute of Applied Radiation Chemistry, Łódź University of Technology, Wroblewskiego 15 93-590, Łódź, Poland; e Viva Biotech Companies, 1 Broadway, Cambridge, MA 02142, USA; f Department of Physical Chemistry, University of Chemistry and Technology, Technická 5, 166 28 Prague, Czech Republic; g Materials Science and Engineering Program, University of California, Riverside, CA 92521, USA

**Keywords:** Dipole, Conformation, Solvation, Transition state, Electret, Onsager reaction field

## Abstract

Electric dipoles are ubiquitous. They affect charge transfer, self-assembly, materials performance, and enzymatic activity. Herein, we demonstrate dipole effects on molecular geometry. An aromatic amide, 5-*N*-amide derivative of anthranilamide (Aaa), assumes two stable conformations with drastically different dipole moments. In non-polar solvents, Aaa exists predominantly as the conformer with the smaller dipole as nuclear Overhauser effect (NOE) and density-functional theory (DFT) analysis reveal. Increasing medium polarity drives the emergence of the other structure with the larger dipole. Splitting of the NMR signals at low temperature is consistent with capturing the two Aaa conformers upon its aggregation. Analysis employing density-functional theory quantifies the dynamics of the equilibrium between the two conformations and how solvent polarity affects it. This synergy between molecular electric dipoles and medium polarity reveals a paradigm for conformational switching.

## 1. Introduction

The ubiquitous nature of electric dipoles places them at the center of a broad range of phenomena of crucial importance [1]. Essential for sustaining life, protein macrodipoles facilitate ion transport through cell membranes [2,3]. Interfacial dipoles define the performance of electric junctions and devices [4-17]. Localized electric fields around molecular dipoles affect enzymatic activity [18-22] and rectify charge transfer (CT) [23-30]. Such localized fields that molecular dipoles and ionic species generate impact optical transitions, as intramolecular electro- chromism, i.e., intramolecular Stark effects, reveal [31-34]. These effects extend to photochemical synthesis, as dipoles affect light-driven chemical transformations [35-38], and evidence for the importance of electrical dipoles in photocatalysis have recently emerged [39-43].

Conformational changes often alter the magnitude and orientation of dipoles [13,24,44-67]. Emerging examples show the opposite paradigm, i.e., dipoles affecting conformational preferences [68-71]. Dipole changes accompanying structural fluctuations, however, do not imply that the dipoles of these structures play deterministic role in their conformational dynamics.

Polyprolines illustrate a classic example of conformational switching along with huge dipole changes that accompany it. Nevertheless, the macrodipole of these poly-*α*-amino acids do not govern their conformational preference. Polyproline forms two stable helical conformers with different electric macrodipoles: (1) tightly packed polyproline type I (PPI) with a large dipole of 4.1 D per residue; and (2) extended polyproline type II (PPII) with a small macrodipole of about 0–1.5 D per residue [24,72]. Changing solvent polarity allows transitioning between PPI and PPII. Aqueous media favors the extended PPII conformer with a small macrodipole but with backbone amides exposed to the solvent [73]. Hydrogen bonding with the solvent enhances the stability of this extended structure [73,74]. Lowering medium polarity drives the formation of PPI that has a large macrodipole but the densely packed hydrocarbon side chains of its residues isolate the amides from the solvating environment [74]. Considering the solvation energy of the dipoles, therefore, an increase in medium polarity should stabilize PPI, rather than PPII [75], which is not the case. That is, localized van der Waals and hydrogen-bonding interactions prevail over the solvation of the polyproline macrodipole for the transition between its PPI and PPII conformers.

The contrast between the examples of dipoles affecting and not affecting molecular conformations warrants a close look at the interplay between the free energy governing structural dynamics. Features that ensure and harness dipole effects on conformational switching provide not only understanding of biochemical phenomena, but also molecular-level design principles for non-native systems, such as biomimetic and bioinspired conjugates, as well as biosensor and actuators.

**Chart 1.**
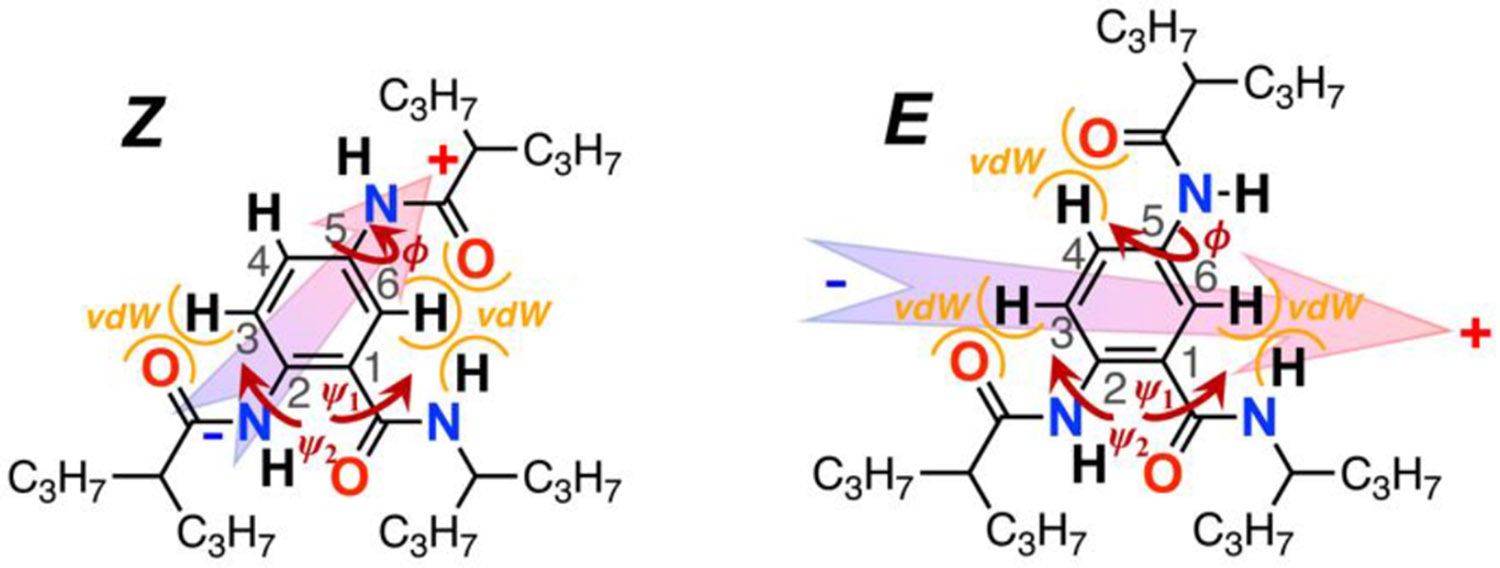
The *Z* and *E* planar conformers of 5-*N*-amidoanthranilamide (**Aaa**), along with their dipoles and sites of van der Waals (vdW) steric hindrance inducing the twists of the amides of the plane of the aromatic ring. The three amides of Aaa are capped with 4-heptyls for improved solubility in organic solvents. For CH_2_Cl_2_, the ground-state permanent electric dipole of the *Z* conformer is 4.7 D, and of the *E* conformer is 11 D; and the angle between the dipoles of the *E* and *Z* conformers is 56°. These trends are the same for all examined solvents with different polarity; and *ϕ*, *ψ*_1_ and *ψ*_2_ are the dihedral angles of rotation of the three amides out of the plane of the aromatic ring.

Herein, we demonstrate how the solvation of the dipole of a building block for bioinspired electrets, i.e., *N*-amidated anthranilamide (**Aaa**), defines the conformation it assumes (Chart 1). Based on the orientation of the amide side chain, **Aaa** assumes two preferred conformations, *E* and *Z*, i.e., **Aaa**^**(E)**^ and **Aaa**^**(Z)**^, respectively, with drastically different dipole moments (Chart 1). The less polar of the two, i.e., the *Z*-conformer, is the more stable and, hence, the predominant structure. An increase in solvent polarity, however, enhances the population of the *E*-conformer, which has a larger dipole moment than the *Z* one. In this case, all amides that define the total dipole of **Aaa** are equally exposed to the solvation media regardless of the assumed geometry. It allows the molecular dipole (rather than the van der Waals and hydrogen-bonding interactions with the solvent) to take precedence and, in synergy with medium polarity, to control the conformational equilibrium.

## 2. Experimental

### 2.1. Materials

The title compound, *N*,*N*′-(2-(heptan-4-ylcarbamoyl)-1,4-phenylene) bis(2-propylpentanamide), **Aaa**, was synthesized in good yields following procedures that we have previously reported [76-78]. The reagents, electrolytes and solvents (with the required grade) were purchased from Fisher Scientific, Millipore Sigma and Cambridge Isotope Laboratories.

### 2.2. Steady-state optical spectroscopy

Steady-state absorption spectra were recorded in a transmission mode using a JASCO V-670 spectrophotometer (Tokyo, Japan); and steady-state emission spectra were measured, also in a transmission mode, with a FluoroLog-3 spectrofluorometer (Horiba-Jobin-Yvon, Edison, NJ, USA) as previously reported [79-82].

### 2.3. Pulse radiolysis

Pulse radiolysis experiments with time-resolved optical absorption detection were carried out at the Institute of Nuclear Chemistry and Technology in Warsaw, Poland. LAE-10 linear electron accelerator, delivering 10 ns pulses with electron energy of about 10 MeV, was employed as a source of ionizing radiation. A low noise 150-W xenon arc lamp E7536 (Hamamatsu Photonics K.K) was used as a probing light source. The respective wavelengths were selected via MSH 301 (Lot Oriel Gruppe) monochromator with resolution ±7.5 nm. The intensity of probe light was measured using PMT R955 (Hamamatsu). The measured signal was digitized using a WaveSurfer 104MXs-B (1 GHz, 10 GS/s, LeCroy) oscilloscope and then forwarded to a PC for further processing. Water filter was used to eliminate the IR radiation that can lead to sample heating. To avoid photodecomposition and photobleaching of the samples, UV cut-off filters were used. Nevertheless, no evidence of photobleaching was found within the time monitored domains. All experiments were performed using flow system with a standard quartz cell (optical path 1 cm) and at temperature set at 22 °C. Details of the pulse radiolysis setup are available elsewhere [83-87].

### 2.4. Cyclic voltammetry

Cyclic-voltammetry measurements are conducted using Reference 600^™^ Potentiostat/Galvanostat/ZRA (Gamry Instruments, PA, U.S.A.), connected to a three-electrode cell, as previously described [88,89]. Anhydrous aprotic solvents with different polarity, such as dichloromethane (CH_2_Cl_2_) and acetonitrile (CH_3_CN), are employed with different concentrations of (*n*-C_4_H_9_)_4_NPF_6_ as a supporting electrolyte. Prior to recording each voltammogram, the sample is extensively purged with argon while maintaining its volume constant by adding more of the anhydrous solvent. The voltammograms are recorded at a scan rate of 50 mV s^−1^.

### 2.5. NMR

The ^1^H NMR spectra were recorded on 600 MHz spectrometers using CDCl_3_ and CD_3_CN as solvents. Chemical shifts were confirmed relative to the solvent peaks. For the aggregation studies, the concertation of **Aaa** was varied between 1 mM and 60 mM [90-93]. For the temperature-dependence studies, the concentration of **Aaa** was kept under 10 mM.

### 2.6. Computational studies

All quantum-chemical calculations, employing density functional theory (DFT), were performed in software package Gaussian 16, revision B.01 [94], employing the range-separated hybrid CAM-B3LYP [95] functional along with the semi-empirical damped D3(BJ) [96] model of dispersion interactions and a split-valence 6-311+G(d,p) basis set. Transition-state conformations were searched for and optimized using relaxed scan of the potential energy. The rigid-rotor harmonic-oscillator model and unscaled fundamental vibration frequencies were used to calculate the thermal contributions to thermodynamic properties of the individual conformational states. Wherever applicable, implicit solvation was considered using the polarizable continuum model (PCM) with the integral equation formalism as implemented in Gaussian [97].

## 3. Results and discussion

### 3.1. Introducing Aaa

**Fig. 1.**
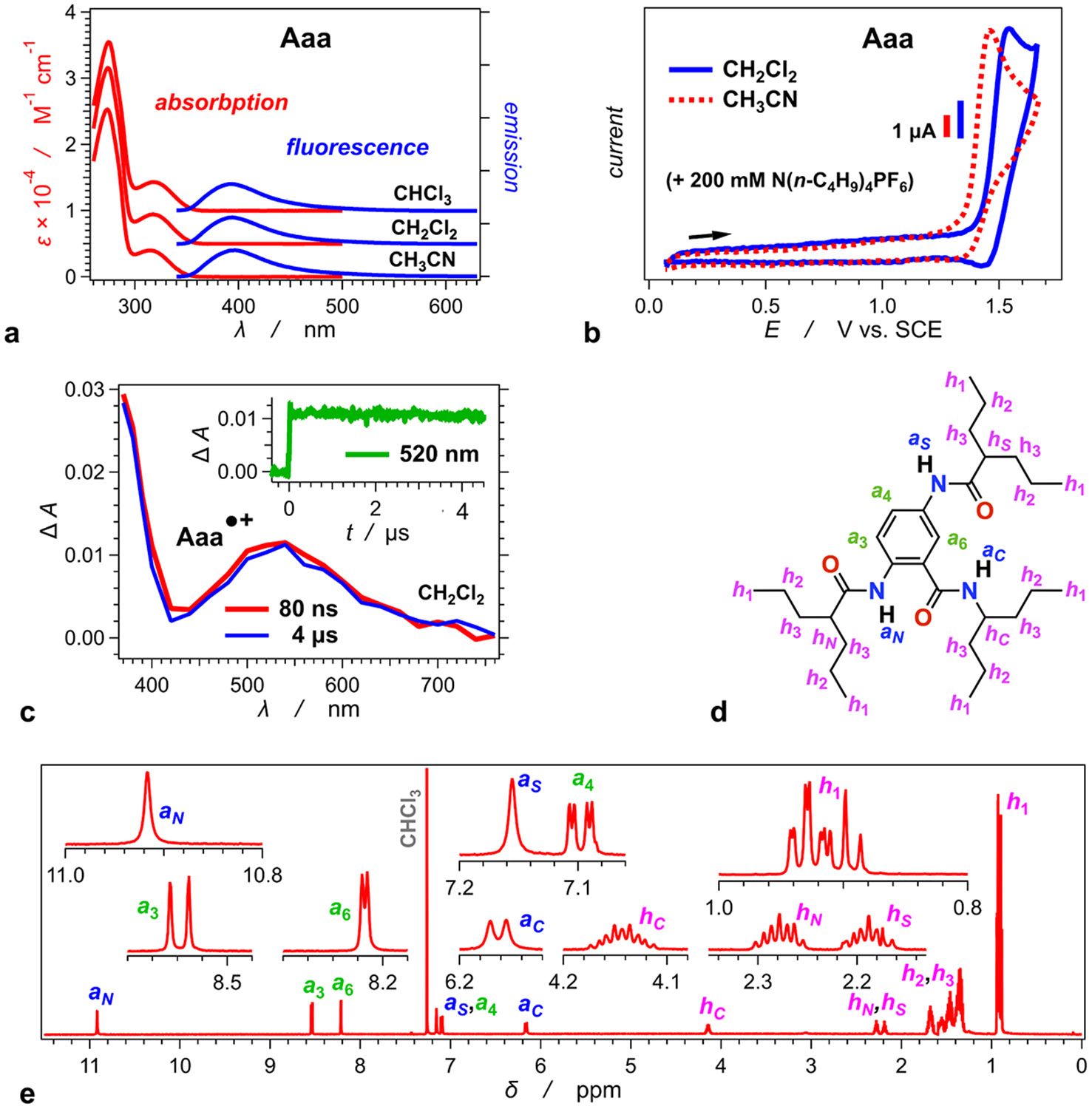
Optical, reducing and NMR properties of **Aaa**. (a) Steady-state optical absorption and emission spectra of **Aaa** in different organic solvents (*λ*_*ex*_ = 325 nm). (b) Cyclic voltammograms of **Aaa** recorded for two aprotic solvents in the presence of 200 mM supporting electrolyte (*v* = 50 mV s^−1^). (c) Absorption spectrum of singly oxidized **Aaa**, i.e., its radical cation, **Aaa**•^+^, obtained from pulse radiolysis measurements of **Aaa** solutions in CH_2_Cl_2_. (d) Structural formula of **Aaa**^**(Z)**^ with the corresponding designation of the protons, i.e., the aromatic protons (*a*_1_, *a*_4_, and *a*_6_), the amide protons (*a*_*C*_, *a*_*N*_, and *a*_*S*_), and the heptyl protons (*h*_1_, *h*_2_, *h*_3_, *h*_*C*_, *h*_*N*_, and *hS*). (e) ^1^H NMR spectrum of **Aaa** (2 mM) in CDCl_3_, along with assignments to the chemical shifts. The insets show details about the features of the different peaks: the amide signals appear as broad singlets (*a*_*N*_ and *a*_*S*_) and doublets (*a*_*C*_); *a*_4_ is a doublet of doublets due to coupling with *a*_3_ and weak meta coupling with *a*_6_; although the signals from the heptyl protons attached to the tertiary carbons are well separated, they appear as multiplets because of the diastereotopic nature of the methylene protons, *h*_3_ (see Supplementary Materials); and the signals from the methyl protons, *h*_1_, appear as three overlapping triplets.

As a residue for anthranilamide bioinspired molecular electrets [28,29,98-101], **Aaa** is a UV absorber exhibiting a broad fluorescence band extending to about 500 nm (Fig. 1a). Its optical properties do not show noticeable dependence on medium polarity precluding formation of excited states with a CT character. Conversely, **Aaa** is a moderately good electron donor undergoing irreversible or partially reversible oxidation around 1.4 V vs. SCE (Fig. 1b). Nevertheless, the lifetime of its radical cation, **Aaa**•^+^, in aprotic solvents extends well into the microsecond time domain (Fig. 1c), which can ensure long-lived CT states. The optical absorption spectrum of **Aaa**•^+^ shows a broad band spreading over the visible region with a maximum at around 540 nm (Fig. 1c).

The three amides of **Aaa** induce a significant permanent electric dipole (Chart 1) [29]. The chemical shifts of the amide protons, extracted from nuclear magnetic resonance (NMR) spectra, provide important conformational information about **Aaa** (see Supplementary Materials for the assignment of the chemical shifts). In deuterated chloroform, the signal from the proton of the *C*-terminal amide, *a*_*C*_, appears around 6.1 ppm; the signal from the proton of the side-chain amide at position 5, *a*_*S*_, is around 7.2 ppm (Fig. 1d,e); and the signal from the proton of the *N*-terminal amide, *a_N_*, is downfield shifted to about 11 ppm (Fig. 1d,e). Since *a*_*C*_ is slightly farther from the aromatic ring than the other two amide protons, it is less deshielded than them. Nevertheless, this deshielding difference, originating from the different distances between the protons and the aromatic ring, cannot account for the 5-ppm gap between the chemical shifts of *a_C_* and *a_N_*. In fact, the *N*-amide protons, *a*_*N*_ and *a*_*S*_, have similar π-conjugation and electronic coupling with the aromatic ring. The difference between their chemical shifts amounts to 4 ppm (Fig. 1e).

The pronounced deshielding of *a*_*N*_ is consistent with strong hydrogen bonding with the oxygen of the neighbouring *C*-terminal amide, which is expected for such anthranilamide structures [76,99,102]. Conversely, *a*_*C*_ and *aS* are not involved in such intramolecular hydrogen bonding, i.e., no hydrogen-bond acceptor in **Aaa** is situated sufficiently close to either of them.

**Fig. 2.**
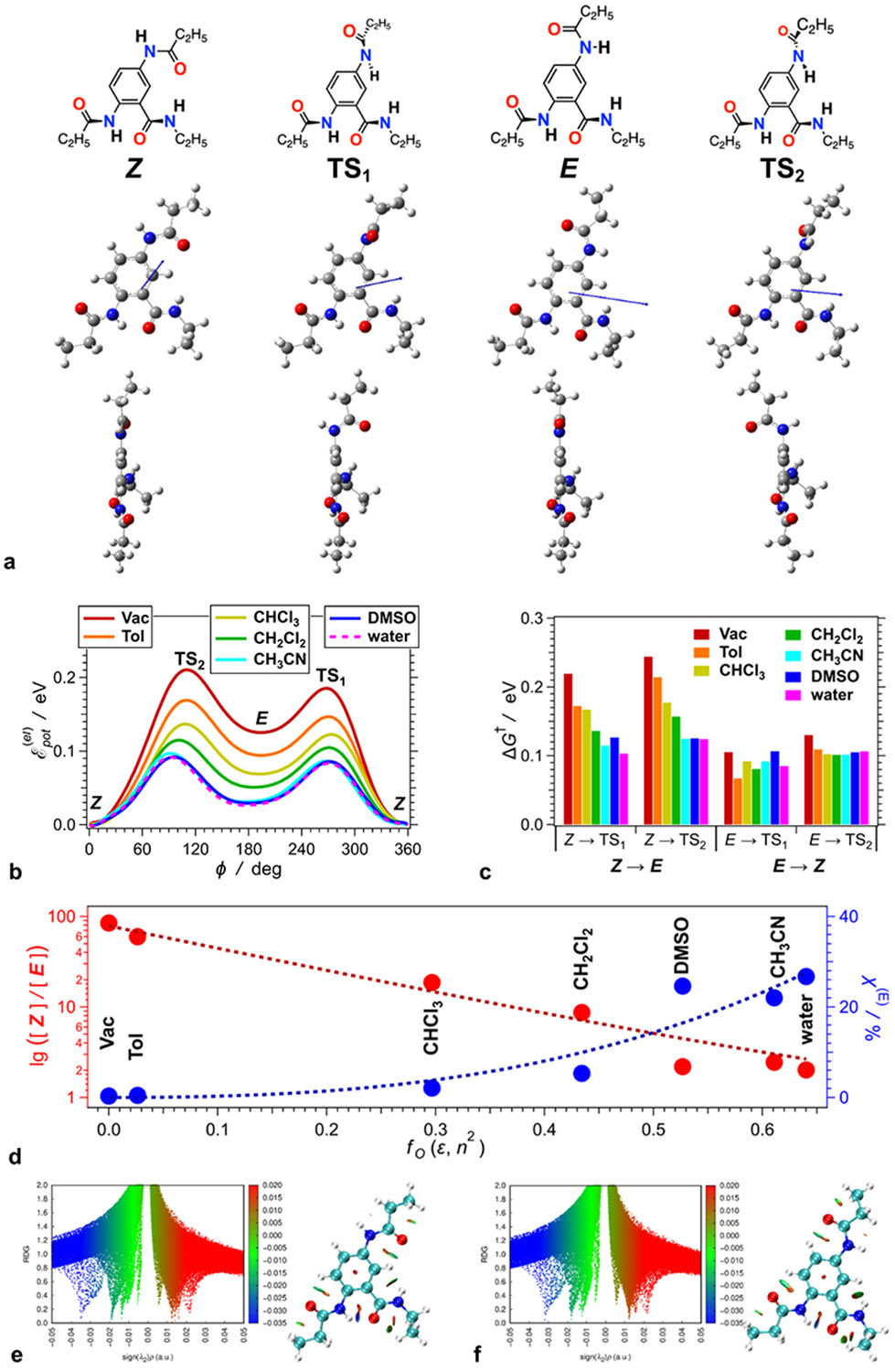
Computational structural analysis of **Aaa** using DFT at the CAM-B3LYP-D3/6–311+G(d,p) level of theory with implicitly introduced solvents. (a) Optimized molecular geometries of stationary points along the trajectory of the torsion of the bond between the nitrogen of the side-chain amide and the carbon at position 5 of the aromatic ring. Upper row: chemical structures drawn to depict the orientation of side-chain amide and the slight twist of the other two amides out of the plane of the aromatic ring; middle row: the optimized structures, along with their dipole moments, viewed orthogonally to the plane of the aromatic ring; and lower row: the same structures viewed along the plane of the aromatic ring (from the *N*-terminus) illustrating the twists of the amide out of the ring plane. The structures are optimized in the presence of implicitly introduced CH_2_Cl_2_. The *E* and *Z* conformers are shown along with the transition states, TS_1_ and TS_2_, for **Aaa**^**(Z)**^ ⥂ **Aaa**^**(E)**^. Because of the sterically induced twist of the *C*- and *N*-terminal amides off the plane of the aromatic ring, the two transition states are not equivalent, i.e., depending on the direction of rotation of the side-chain amide, the transformation between **Aaa**^**(Z)**^ and **Aaa**^**(E)**^ proceeds through a different transition state. (b) Electronic potential energy profiles along the torsion mode enabling the transition between **Aaa**^**(Z)**^ and **Aaa**^**(E)**^, in the gas phase (vacuum) and in the presence of six implicitly introduced solvents with different polarity. The torsion angle ϕ represents the angle between the plane of the side-chain amide and the plane of the aromatic ring. (c) Transition-state energies, Δ*G*^†^, represented as the Gibbs free energies required to reach TS_1_ and TS_2_ from the “stable” **Aaa**^**(Z)**^ and **Aaa**^**(E)**^ structures at 25 °C. (d) Estimated ratios between the mole fractions, *χ*, of **Aaa**^**(Z)**^ and **Aaa**^**(E)**^, i.e., $\frac{\left[Z\right]}{\left[E\right]}=\text{exp}\left(-\frac{\Delta G_{E\to Z}}{k_{B}T}\right)$ for 25 °C, along the relative fraction of the less-stable *E* (%) conformer, i.e., $\chi^{\left(E\right)}(\%)=100\;[E]\;{\left([Z]+[E]\right)}^{1}=100\frac{\left[E\right]}{\left[Z\right]}{\left(1+\frac{\left[E\right]}{\left[Z\right]}\right)}^{-1}$. (e,f) Analysis of intramolecular non-covalent interactions (NCI) in (e) **Aaa**^**(Z)**^ and (f) **Aaa**^**(E)**^ in chloroform. Data are shown in terms of the reduced density gradient (RDG) and the sign of the second density Hessian eigenvalue (*λ*_2_), with values presented in atomic units (a.u.). The blue isosurfaces on the structures, between the *N*- and *C*-terminal amides, represent the attractive region of the reduced-density plot in the interval of sign(*λ*_2_)•*ρ* from −0.4 a.u. to −0.3 a.u. corresponds to hydrogen bonding. The green isosurfaces, −0.2 a. u. < sign(*λ*_2_)•*ρ* < −0.0 a.u., emerging between carbonyl oxygens and nearby aromatic or aliphatic hydrogens, are attributed to dispersion attractive interactions. These attractive interactions are closely coupled with repelling interactions between non-hydrogen atoms (orange and red isosurfaces).

The hydrogen bond between the *C*-terminal and *N*-terminal amides holds them almost in a coplanar conformation (Fig. 2a). The side-chain amide at position 5, on the other hand, can rotate around its bond with the aromatic ring, which results in two preferred planar conformations, *Z* and *E* (Chart 1, Fig. 2a,b), stabilized by the extended π-conjugation.

Computational analysis confirms the hydrogen bond between the *C*-terminal and *N*-terminal amides. Analysis of intramolecular non-covalent interactions (NCI) in the framework of the quantum theory of atoms in molecules (QTAIM) reveals that the only hydrogen bonding in **Aaa** is between the hydrogen *a_N_* of the *N*-terminal amide and the carbonyl oxygen of the *C*-terminal amide (Fig. 2e,f, S10). This finding agrees well with the NMR results. Furthermore, the NCI analysis reveals that this intramolecular hydrogen bond wanes as the solvent polarity increases. This effect is stronger for the *Z* than the *E* species.

### 3.2. Amides are twisted out of the ring plane

Electronic π-conjugation of the amides with the aromatic ring warrants preference for planar conformation. Conversely, steric hindrance with the adjacent aromatic protons slightly twists the amides off the plane of the ring (Chart 1, Fig. 2a). Specifically, van der Waals contacts with aromatic proton 6, *a*_6_, induces a 23° dihedral angle between the *C*-terminal amide and the ring in the *Z* conformer in the gas phase. Similar steric hindrance with proton 3, *a*_3_, twists the *N*-terminal amide 6° out of the ring plane. The *N*-terminal and side-chain amides have similar electronic coupling and steric interactions with the aromatic ring (Tables 1, S5).

Considering the length of the covalent bonds and the size of the atoms involved in these van der Waals interactions suggests that the N-terminal amide should twist out of plane to a larger extent than the *C*-terminal one. The carbonyl oxygen, coming in contact with *a*_3_, is larger than amide hydrogen, *a*_*C*_, that is in contact with *a*_6_. Furthermore, the C=O bond is longer than the N–H one that, in planar conformation, ought to place the amide oxygen closer to *a*_3_ than *a_C_* to *a*_6_ (Fig. 1d). These structural features warrant more pronounced steric hindrance of the aromatic protons with the *N*-terminal than the *C*-terminal amide.

Nevertheless, the *C*-terminal amide is twisted at a larger angle out of the aromatic-ring plane than the *N*-terminal one. The reason for this discrepancy appears to originate from differences in the electronic contribution to the stabilization of the planarity of **Aaa**. The π_nb_ and n frontier orbitals of amides have nodes at their carbonyl carbons [103,104]. Therefore, *C*-amides (attached to aromatic rings via their carbons) have relatively small mesomeric (i.e., resonance) effect as functional groups [105,106]. Conversely, *N*-amides (attached via their nitrogens) tend to exhibit strong π- conjugation with aromatic rings and act as moderate electron-donating substituent [105-107].

The highest occupied molecular orbitals (HOMO) of **Aaa** expand over the *N*-terminal and the side-chain amides at position 2 and 5, respectively, but not over the *C*-terminal amide at position 1 (Figs. S4 and S5). It illustrates pronounced π-conjugation through the nitrogens and no significant conjugation through the carbonyl carbons. The ellipticity of the bonds between the amides and the aromatic ring confirms this conjugation pattern (Figs. S7 and S8). Natural bonding orbital (NBO) analysis reveals that the lone electron pairs on the nitrogens of the *N*-terminal and side-chain amides strongly delocalize not only over the carbonyls, but also toward the aromatic ring. Conversely, the lone pair on the *C*-terminal amide delocalizes only over the carbonyl, does not extend to the aromatic ring, and exhibits non-negligible hyper conjugation with the aliphatic moiety (see Supplementary Materials).

Therefore, the *N*-terminal and side-chain amides in **Aaa** experience stronger π-conjugation with the ring and more pronounced electronic stabilization of their planar conformations than the *C*-terminal one. It is consistent with the *C*-terminal amide assuming larger twist off the plane of the ring than the other two amides of **Aaa**. Even though hydrogen bonding stabilizes the planar conformation of the *C*-terminal amide, it is still twisted at a larger angle than the side-chain amide, which is not hydrogen bonded (Tables 1, S5). These findings indicate that, for the preferred **Aaa** conformations, the electronic effects dominate over the steric hindrance.

### 3.3. The two conformers have different dipoles

The ordered orientation of the *C*-terminal and *N*-terminal amides, locked in an almost planar conformation by the hydrogen bond between them, provides a constant contribution to the **Aaa** electric dipole. The amide at position 5, however, can rotate around its bond with the aromatic ring. This rotation changes the amide orientation and considerably affects the total dipole of **Aaa** (Fig. 2a). The π-conjugation that extends over this *N*-amide at position 5, however, stabilizes preferentially the two structures with enhanced planarity, i.e., an *E* and a *Z* conformer (Fig. 2b).

Still, the steric hindrance between the oxygen of this amide and proton 6, *a*_6_, of the aromatic ring (Fig. 1d, Chart 1) induces 7.4° dihedral angle in **Aaa**^**(Z)**^ for CH_2_Cl_2_ (Table 1). Similarly, steric hindrance with proton 4, *a*_4_, in **Aaa**^**(E)**^ (Fig. 1d, Chart 1) induces 8.4° dihedral angle (Table 1).

A principal difference between the *E* and *Z* conformers is the magnitude and the orientation of their electric dipole moments. The carbon–nitrogen bonds of amides are partially π-conjugates and relatively rigid, existing as *trans* or *cis* conformers. In **Aaa** all amides remain in the *trans* geometry, and the change in the dipole originates from a twist around the carbon–nitrogen bond between the side-chain *N*-amide and the aromatic ring, i.e., from the transition between the planar *E* and *Z* conformers (Chart 1). The dipole of **Aaa**^**(E)**^ is more than twice larger than the dipole of **Aaa**^**(Z)**^ (Table 1). The angle between the dipoles of **Aaa**^**(E)**^ and **Aaa**^**(Z)**^ is 54° for toluene and increases to 57° for polar solvents, such as acetonitrile (Table 1). The magnitude of a dipole of a single amide can vary between about 3.5 D and *>*10 D, depending on the polarity of the solvating media [108]. Therefore, it is not surprising that the orientation of the amide at position 5 can have such an enormous effect on the total dipole of **Aaa**, and on its overall electronic properties. Switching between *E* and *Z* conformations, for example, affects the π-conjugation of the aromatic ring with the nitrogen at position 2, and the hydrogen bond between the *C*- and *N*-terminal amides. (Figs. S7 and S9).

### 3.4. Effects of solvent polarity

Computationally, indeed, we can implicitly introduce a wide variety of solvents, and we do (Table 1). Implicit solvating media, however, do not account for specific intermolecular interactions with the solute, **Aaa**. Nevertheless, explicit implementation of hydrogen-bonding solvents reveals distortions of anthranilamide structures [98,109]. Our experimental studies of **Aaa**, therefore, avoid protic solvents, such as alcohols, and hydrogen-bond-accepting solvents containing functional groups such as carbonyls, ethers and sulfoxides. Solubility of **Aaa** in different solvents presents another important consideration. For example, while **Aaa** is not soluble in water, which precludes its experimental studies in aqueous media, introducing water in the computational analysis allows examining the effects of such polar, but not too polarizable, solvating media (Fig. 2b-d, Table 1).

**Fig. 3.**
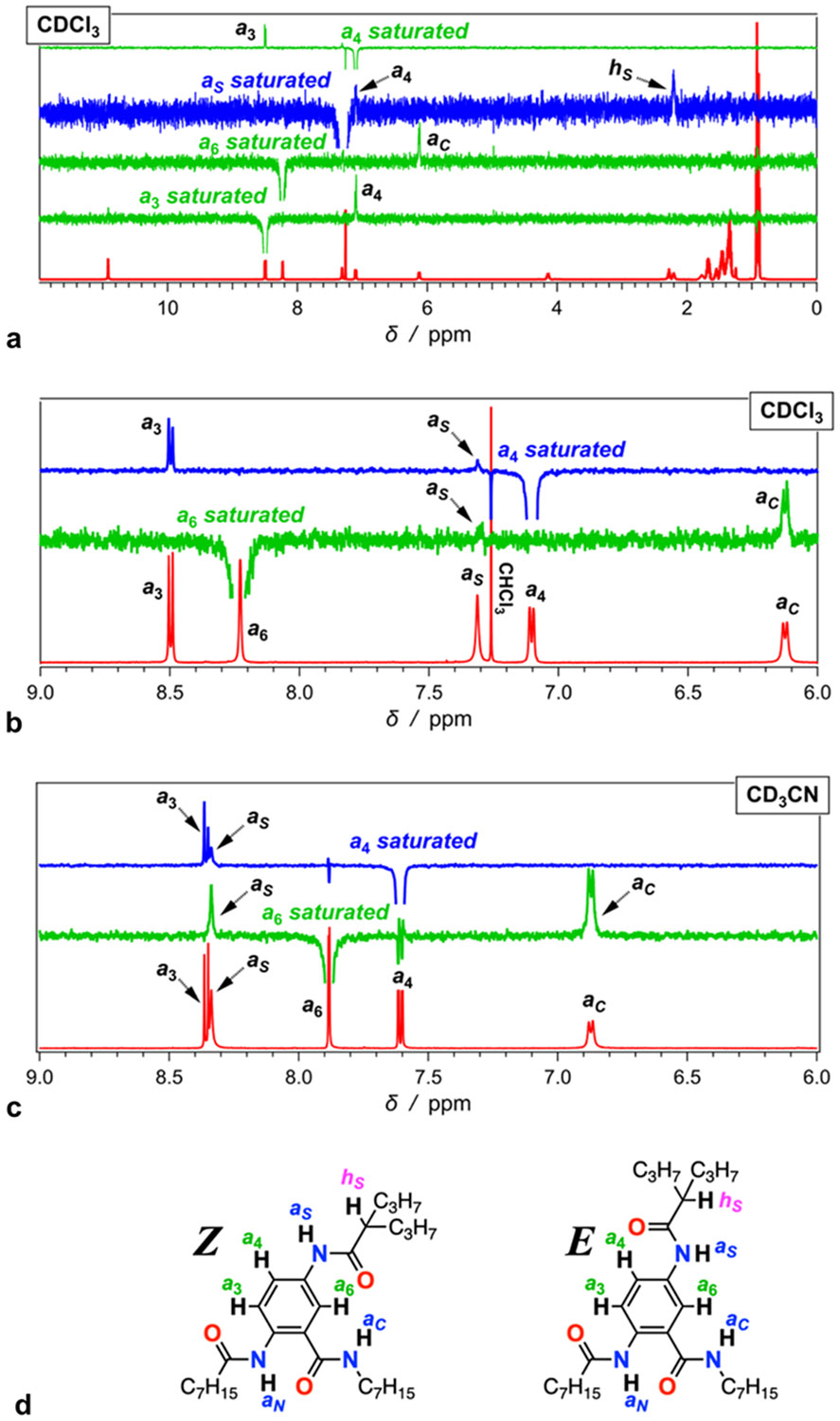
Nuclear Overhauser effect (NOE) analysis of **Aaa**. (a-c) One-dimensional (1D) NOE spectra obtained upon selective excitation of different protons and saturation of their signals. The corresponding ^1^H NMR spectra are shown in red on the bottom of each graph. (a,b) Spectra of 15 mM **Aaa** in deuterated chloroform, CDCl_3_, with (b) focusing on the aromatic and amide; and (c) spectra of 7 mM **Aaa** in deuterated acetonitrile, CD_3_CN. (d) Structures of the *E* and *Z* conformers of **Aaa** with the designations of the aromatic and amide protons referenced in the NOE spectra.

As implemented by ^1^H NMR, nuclear Overhauser effect (NOE) can prove informative about the preferred orientation of the side-chain amide of **Aaa**. Saturating the *a*_6_ signal enhances predominantly the signal from *a_C_*, along with some residual enhancement of *a_S_* (Fig. 3a,b). Similarly, saturating *a*_4_ enhances the signal of *a*_3_ and partially that of *a*_*S*_ (Fig. 3a,b). These results confirm the proximity between *a*_6_ and *a_C_* protons, as well as between *a*_3_ and *a*_4_. They do not provide unequivocal evidence, however, if *a*_*S*_ is closer to *a*_4_, i.e., *Z*-conformer, or to *a*_6_, i.e., *E*conformer. Saturation of *a_S_*, on the other hand, shows some enhancement of the *a*_4_ signal, along with that of the heptyl *h*_*S*_ proton, without a detectable increase in the *a*_6_ signal (Fig. 3a). Such a through-space correlation between *a*_*S*_ and *a*_4_ suggests a preference for the *Z*-conformer when **Aaa** is in chloroform (Fig. 3d).

Conversely, the NOE spectra of **Aaa** in acetonitrile show different trends. Saturating *a*_6_ leads to similar increases in the *a*_*S*_ and *a*_*C*_ signals (Fig. 3c), which contrasts the findings for chloroform where the enhancement of *a*_*S*_ upon saturating *a*_6_ is marginal (Fig. 3b). Also, saturating *a*_4_ of **Aaa** in CD_3_CN enhances the overlapping signals of *a*_*S*_ and *a*_3_ (Fig. 3c). These findings for acetonitrile indicate abundant presence of both conformers, **Aaa**^**(E)**^ and **Aaa**^**(Z)**^. That is, while in low-polarity media favor the *Z*-conformer, increasing solvent polarity drives the emergence of **Aaa**^**(E)**^ (Fig. 3d).

An increase in medium polarity increases the dipoles of both **Aaa** conformers, which is consistent with enhancing the Onsager reaction field in the solvation cavity [75,108]. Furthermore, medium polarity stabilizes dipolar species, i.e., lowers their energy. This polarity-induced stabilization is more pronounced for the *E* conformer, **Aaa**^**(E)**^, that has a larger dipole than **Aaa**^**(Z)**^ (Table 1). That is, while **Aaa**^**(Z)**^ is more stable than **Aaa**^**(E)**^, the difference between their energies decreases with increasing solvent polarity, e.g., Δ*G*_E→Z_ = Δ*G*_Z_ – Δ*G*_E_, is about 4 *k*_*B*_*T* for toluene, and about 0.9 *kBT* for acetonitrile (Table 1). Therefore, at room temperature the estimated population of **Aaa**^**(E)**^ in toluene is about 0.5 %. In chloroform, it increases to 2 % and reaches 22 % in acetonitrile (Fig. 2d).

Dipoles originate from separating the overlapping distributions of the negative and positive charges. An increase in medium polarity favors such charge separation. In the case of **Aaa**, the rotation of the side-chain amide at position 5 around the C-N bond, connecting it with the aromatic ring, has a pronounced effect on this separation between the centers of masses of the positive and negative charges. Therefore, medium polarity affects the ratio between the populations of conformers with different orientation of the amide side chain since they have different separation between the negative and positive charge densities.

### 3.5. On the hunt for the E conformer

**Fig. 4.**
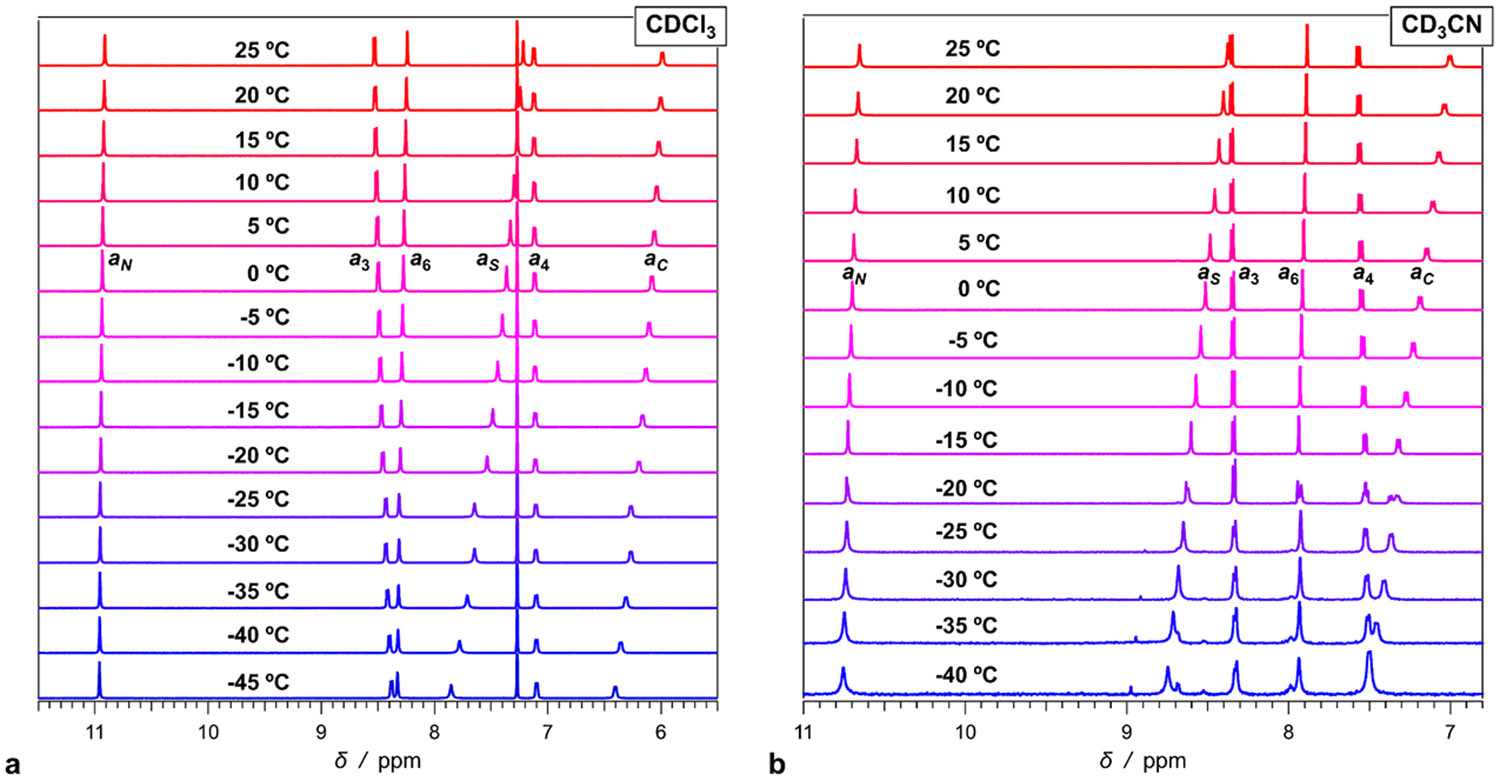
Temperature dependence of the ^1^H NMR spectra of **Aaa** (7 mM) depicting the signals from the aromatic and the amide protons. (a) Spectra of 7 mM **Aaa** in deuterated chloroform, CDCl_3_, recorded at temperatures ranging from −45 to 25 °C. The solvent peak, i.e., of the residual CHCl_3_, appears at 7.27 ppm. (b) Spectra of 12 mM **Aaa** deuterated acetonitrile, CD_3_CN, recorded for temperatures ranging from −40 to 25 °C. The melting point of CD_3_CN is −46 °C, which precludes further lowering the temperature of these samples where peak splitting emerges.

If the transition rates between the two conformers are slow enough, NMR spectra can reveal signals from **Aaa**^**(E)**^ and **Aaa**^**(Z)**^. For each proton of **Aaa** in CDCl_3_, the ^1^H NMR spectrum shows only a single well-defined peak depicting the multiplicity splitting expected from the corresponding through-bond and meta *J*-coupling (Fig. 1e). Lowering the temperature to −45 °C induces: (1) pronounced downfield shifts of the *a_S_* and *a*_*C*_ amide protons, (2) slight downfield shifts of the amide *a*_*N*_ and the aromatic *a*_6_ protons, and (3) upfield shifts of the aromatic *a*_3_ and *a*_4_ protons (Fig. 4a). At low temperatures, while spectral broadening becomes apparent, we do not observe splitting of any of the peaks.

This finding is consistent with: (1) low energy barriers for the transition between the *E* and *Z* conformers even at −45 °C, producing **Aaa**^**(E)**^ ⥂ **Aaa**^**(Z)**^ interconversion that is faster than the time scales for recording the NMR spectra; and (2) small abundance of the **Aaa**^**(E)**^ conformer in chloroform, i.e., about 2 %, that may prove undetectable on the ^1^H NMR spectra. The downfield and upfield shifts, on the other hand, most likely originate from aggregation of **Aaa** at low temperature. An increase in **Aaa** concentration at room temperature results in similar NMR shifts (Fig. S3). In fact, the magnitude and direction of the shifts, which lowering the temperature to −20 °C induces, are similar the shifts at room temperature obtained from an increase in **Aaa** concentration to 60 mM.

An increase in solvent polarity increases the population of **Aaa**^**(E)**^ to above 5 % (Fig. 2d), which should make it detectable for NMR. At room temperature, the ^1^H NMR spectrum of **Aaa** in CD_3_CN shows only a single peak for each of the protons (Fig. 4b). Lowering the temperature induces downfield shifts of *a*_6_ and the amide protons, i.e., similar to what we observe for CDCl_3_. In addition to the peak broadening, as the temperature drops below −20 °C, extra signals from the amide *a*_*S*_ and the aromatic *a*6 proton start to emerge (Fig. 4b).

For two states, **A** and **B**, at equilibrium that exchange with rate constants *k*_**A**→**B**_ and *k*_**B**→**A**_, Gutowsky and Holm derived an expression for the Lorentzian NMR line shapes in frequency (*ν*) domain based on the Bloch equations [110-112]:

(1a) $$I(\nu )=\frac{\gamma H_{1}M_{0}}{2\pi }\times \frac{Q(\nu )R(\nu )+P(\nu )\left(1+\frac{\tau }{T_{2}}\right)}{{\left(P(\nu )\right)}^{2}+{\left(R(\nu )\right)}^{2}}$$

where *T*_2_ is the time constant of the transverse (spin–spin) relaxation; *τ* is related to the exchange rate constants, *τ* = ((*k*_**A**→**B**_ *k*_**B**→**A**_)(*k*_**A**→**B**_^−1^ + *k*_**B**→**A**_^−1^))^−1^; *γ* is the gyromagnetic ratio; **H**_1_ is the auxiliary magnetic field; and **M**_0_ is the static nuclear magnetization at thermal equilibrium.

The functions *P*(*ν*), *Q*(*ν*) and *R*(*ν*) depend on the mole fractions of **A** and **B**, i.e., *χ*_**A**_ and *χ*_**B**_, and the resonance frequencies of their nuclei, i.e., *ν*_**A**_ and *ν*_**B**_ [110,112]:

(1b) $$P(\nu )=\frac{1}{T_{2}}+\tau \left({\left(\frac{1}{T_{2}}\right)}^{2}-{\left(\frac{\nu_{A}+\nu_{B}}{2}-\nu \right)}^{2}+{\left(\frac{\nu_{A}-\nu_{B}}{2}\right)}^{2}\right)$$

(1c) $$Q(\nu )=\tau \left(\frac{\nu_{A}+\nu_{B}}{2}-\nu -\frac{\left(\chi_{A}-\chi_{B})(\nu_{A}-\nu_{B}\right)}{2}\right)$$

(1d) $$R(\nu )=\frac{\left(\chi_{A}-\chi_{B})(\nu_{A}-\nu_{B}\right)}{2}+\left(\frac{\nu_{A}+\nu_{B}}{2}-\nu \right)\left(1+\frac{2\tau }{T_{2}}\right)$$

While $k_{A\to B}=\chi_{B}\;\tau^{-1}$ and $k_{B\to A}=\chi_{A}\;\tau^{-1}$ [110] the Eyring theory relates these rate constants to the transition-state enthalpy, Δ*H* ^†^, and entropy, Δ*S* ^†^, at different thermal energy, *k_B_T* [113]:

(2) $$k_{A\to B}=\kappa \frac{k_{B}T}{h}e^{-\frac{\Delta H_{A\to B}^{†}}{k_{B}T}}e^{-\frac{\Delta S_{A\to B}^{†}}{k_{B}}}$$

Assuming Δ*H* ^†^ and Δ*S* ^†^ are constant within the implemented temperature range, allows using eq. 1 and 2 for global fits (GFs) to extract kinetic information from the recorded NMR spectra. When setting the transmission coefficient, *κ*, to unity, the GF analysis of the spectra recorded between −40 and −25 °C produces Δ*H*^†^ = 0.18 eV and Δ*S*^†^ = −1.9 × 10^−3^ eV K^−1^, indicating a relatively large entropic contribution to the transition-state energy, which at room temperature extrapolates to Δ*G*^†^(20 °C) = 0.72 eV. Lowering *κ* to 0.3 does not considerably affect the outcomes from the GF analysis, yielding Δ*H*^†^ = 0.16 eV, Δ*S*^†^ = −1.8 × 10^−3^ eV K^−1^ and Δ*G*^†^(20 °C) = 0.69 eV.

Nevertheless, this analysis warrants a great deal of caution. First, the temperature-induced shifts of the NMR signals suggest that aggregation is most likely involved in the cooling-induced emergence of the small peaks. Even if these spectral patterns represent the **Aaa**^**(E)**^ and **Aaa**^**(Z)**^ populations trapped in slow interexchange, the addition of aggregation equilibria precludes the quantitative validity of the two-state model (Eqs. (1) and (2)). Second, the temperature range of the analysis is quite narrow, which brings inherent uncertainty in the extraction of Δ*S*^†^ as Eq. (2) implies when rearranged to accommodate linear plots of ln(*k*_**A**→**B**_ *T*^−1^) vs. *T*^−1^.

Resorting to *in silico* analysis allows to gain quantitative understanding of the dynamics of the interexchange between the **Aaa**^**(E)**^ and **Aaa**^**(Z)**^ conformers. Scans involving the rotation of the side-chain amide at position 5 around its bond with the aromatic ring reveal two energy barriers between the **Aaa**^**(E)**^ and **Aaa**^**(Z)**^ conformers (Fig. 4b). The structures with the side-chain amide orthogonal to the plane of the ring, i.e., with torsion angles of about 90° and 270°, represent the two transition states (Fig. 4a). The slight off-plane twists of the *C*- and *N*-terminal amides at positions 1 and 2, respectively, along with the variations in the torsion of the alkyl chains, induce a small difference between the two energy barriers.

Implicit implementation of solvents reveals dependence of the transition-state energies on the medium polarity. In addition, it also affects the side-chain dihedral angle, ϕ, of the transition states, TS_1_ and TS_2_ (Fig. 2a). In nonpolar media, the transition states do not assume truly orthogonal conformations, e.g., ϕ of TS_2_ in toluene is about 105°. The ellipticity of the bond between the side-chain amide and the aromatic ring confirms partial π-conjugation even in the transition states (Fig. S7). The most polar among the considered solvents, i.e., water, pushes the torsion of the side chain in the transition state geometry close to 90°. While transferring from water to toluene nearly doubles the transition-state Gibbs energy for transforming **Aaa**^**(Z)**^ to **Aaa**^**(E)**^, it increases Δ*G*^†^ for the reverse step, **Aaa**^**(E)**^ → **Aaa**^**(Z)**^, only by about 18 % (Fig. 2c). Thus, medium polarity stabilizes the transition states and the *E* conformer to a similar extent, which is considerably more than the stabilization of **Aaa**^**(Z)**^. This finding is consistent with the large dipoles of the transitions states at the two energy barriers that are more comparable to the dipole of **Aaa**^**(E)**^ than of **Aaa**^**(Z)**^ (Table 1).

The enthalpic contributions to the barriers, Δ*H*^†^, for the **Aaa**^**(Z)**^ ⇄ **Aaa**^**(E)**^ transformations follow similar trends to those for the transitionstate Gibbs energies, Δ*G*^†^. The computational uncertainty of Δ*H*^†^ determined with this particular level of theory amounts to 15–20 % [114], rendering direct quantitative comparison between barriers for solvents with similar polarity challenging. The interplay of the barrier heights with the variation of the relative energies of **Aaa**^**(E)**^ further blurs such comparisons.

The results from this study reveal the trends regarding the effects of solvent polarity on the barrier heights and the relative conformational energies. The computed Δ*G*^†^ barriers for the **Aaa**^**(Z)**^ → **Aaa**^**(E)**^ transitions in acetonitrile amount to about 120 meV (Fig. 2c), which is six times smaller than the estimates for Δ*G*^†^ obtained from the NMR studies. For the same transitions, the theoretical calculations yield −1.6 × 10^–4^ eV K^−1^ for the entropic contributions to the barriers, Δ*S*^†^, which is an order of magnitude smaller than the experimental estimates. Hence, despite some of their uncertainties, the computational approaches predict quite low energies for the transition states and transition rates that are orders-of-magnitude faster than the NMR timescales. As Eq. (2) reveals, for example, considering the 120-meV activation energy for the *Z*-to-*E* transition in acetonitrile (Fig. 2c) and assuming a transmission coefficient as small as 0.1, result in a rate constant, *k*_*Z*→*E*_, that is in the order of 1.2 × 10^9^ s^− 1^ for – 40 ° C, rendering the process way too fast for NMR measurements to detect separate signals from the two conformers during the equilibrium exchange between them.

While such nanosecond exchange rates are extremely fast for NMR detection, they are still sufficiently slow to yield heterogeneous kinetics of a wide range of processes, such as picosecond photoinduced charge transfer [29].

These considerations indicate that the conformational transitions cannot be solely responsible for the observed temperature-induced changes in the NMR spectra (Fig. 4b). Indeed, lowering the temperature does not sufficiently slow the **Aaa_Z_** ⇄ **Aaa_E_** exchange for the observed signal splitting to emerge. Such suppression of the exchange dynamics ought to originate from other processes, such as aggregation, which in its own turn is enhanced by lowering the temperature. Furthermore, changes in the NMR shifts upon lowering of the temperature are consistent with aggregation (Fig. S3). Conversely, the acetonitrile medium produces signal splitting, while chloroform does not (Fig. 4). It is consistent with the polar media shifting the equilibrium toward the conformer with a large dipole and enhancing its population sufficiently to be NMR detectable upon aggregation. As informative as the low-temperature NMR spectra are as evidence for the presence of two major **Aaa** structures, they do not provide quantitative information about the energetics of the dynamic equilibrium between the two conformers. In synergy, computational analysis elucidates the effects of medium polarity on the conformational exchange kinetics.

## 4. Conclusions

When two conformers have different electric dipoles, the polarity of the solvating medium can control the equilibrium between them. Polar solvents stabilize the conformer with a large dipole and enhance its population. This behavior is opposite to the solvation effect on the equilibrium between PPI and PPII, for example, where polar media stabilize the structure with a small or no net macrodipole but with the backbone amides exposed to the solvent [74,115]. That is, the effects of localized solvation are dominant for polyproline. In both conformers of conjugates like **Aaa**, on the other hand, the polar amide groups are equally exposed to the solvent, rendering energy differences originating from localized interaction with the solvating media negligible. It allows the molecular macrodipoles, rather than the local van der Waals solvation interactions, to govern the effects of medium polarity on the conformational equilibria. These results for **Aaa** present key considerations and paradigm for molecular switches, supramolecular designs and energy-conversion systems.

## Supplementary Material

1

## Figures and Tables

**Table 1:** Dipole moments and twisting of the side-chain of the *Z* and *E* conformers of **Aaa** in solvents with different polarity.^a^

Solvent^b^	ϕ^(Z)^/deg^c^	ϕ^(E)^ deg^c^	*μ*^(Z)^/D^d^	*μ*^(E)^/D^d^	*θ*μ/deg^e^	Δ*G*_E→Z_/ meV^f^
Vac	−12.8	3.1	3.73	7.78	53	−114
Tol	−12.5	1.5	4.28	9.27	54	−105
CHCl_3_	−11.2	1.2	4.52	10.2	55	−75.1
CH_2_Cl_2_	−8.9	0.9	4.65	10.7	56	−55.5
CH_3_CN	−6.2	−2.0	4.79	11.3	57	−23.0
DMSO	−6.0	−2.6	4.81	11.4	57	−20.2
Water^g^	−5.7	−3.4	4.82	11.5	57	−18.0

Note: Chart 1 Footnote Found Same Para Kindly check.

a From DFT calculations at the CAM-B3LYP-D3/6–311+G(d,p) level of theory.

b In the gas phase (Vac) and implicitly implemented solvents: toluene (Tol), chloroform (CHCl_3_), dichloromethane (CH_2_Cl_2_), acetonitrile (CH_3_CN), dimethylsulfoxide (DMSO), and water.

c Dihedral angles between the side-chain amide, attached to carbon 5, C^(5)^, and the aromatic ring, i.e., for the *Z* conformer, ϕ^(Z)^ is defined by C^(6)^–C^(5)^–N–C (O) bonds, and for the *E* conformer, ϕ^(E)^ is defined by C^(4)^–C^(5)^–N–C(O) bonds (Chart 1, Fig. 1d).

d Magnitude of the ground-state dipoles, *μ* = |**μ**|, of the *Z* conformer, *μ*^(Z)^, and the *E* conformer, *μ*^(E)^.

e Angle between the orientations of the dipoles of the *Z* conformer, **μ**^(Z)^, and the *E* conformer, **μ**^(E)^.

f The **Aaa** derivative capped with 4-heptyls (Chart 1) is not soluble in water. Nevertheless, the computational implementation of this solvent is for comparison with the less polar organic media.

g Gibbs free energy of transitioning the *E* to the *Z* conformer, Δ*G*_E→Z_ = Δ*G*_Z_ – Δ*G*_E_.

## Data Availability

Data will be made available on request.

## Appendix A. Supplementary data

Supplementary data to this article can be found online at https://doi.org/10.1016/j.jphotochem.2025.116362.

## Glossary

Aaa: *N,N′*-(2-(heptan-4-ylcarbamoyl)-1,4-phenylene)bis(2-propylpentanamide), i.e., 5-*N*-amide derivative of anthranilamide (Chart 1)
CT: charge transfer
DFT: density-functional theory
DMSO: dimethyl sulfoxide
GF: global fit
HOMO: highest occupied molecular orbital
LUMO: lowest unoccupied molecular orbital
NCI: non-covalent interactions
NBO: natural bonding orbital
NMR: nuclear magnetic resonance
PCM: polarizable continuum model
PPI: polyproline type I
PPII: polyproline type II
QTAIM: quantum theory of atoms in molecules
RDG: reduced density gradient
Tol: toluene
Vac: vacuum or “the gas phase”
vdW: van der Waals
