# Supplementary material for: Dipoles affect conformational equilibrium
Source: J Photochem Photobiol A Chem. Author manuscript; Available in PMC 2026 May 29. (PMC13218642; doi:10.1016/j.jphotochem.2025.116362)
Supplement: 1 [file NIHMS2172104-supplement-1.pdf]

Dipoles Affect Conformational Equilibrium
(Supplementary Material)

Eli M. Espinoza, J. Omar O'Mari, James B. Derr, Mimi Karen Billones, John A. Clark, Maryann Morales
Tomasz Szreder, Bing Xia, Javier Ceballos, Ctirad Červinka, and Valentine I. Vullev

<u>Table of contents</u>

Materials…… S2
Methods…… S2
Pulse radiolysis…… S4
NMR analysis…… S4
  Assignment of chemical shifts…… S4
  Concentration dependence of the chemical shifts…… S6
  Temperature dependence…… S6
Computational studies…… S7
  Bond ellipticity…… S9
  Natural bonding orbital (NBO) analysis…… S10
  Evaluation of the hydrogen bonding…… S12
  Energy minima and transition states…… S15
  Transition-state energies…… S18
  Implicit solvent models…… S18
SM References…… S20

Materials

All chemicals were used as received unless otherwise noted. The principal compound, *N*,*N'*-(2-(heptan-4-ylcarbamoyl)-1,4-phenylene)bis(2-propylpentanamide) (**Aaa**), was synthesized in three steps (Scheme S1) [1, 2], following established procedures for making anthranilamide derivatives.[3, 4] The starting material, 5-amino-2-nitrobenzoic acid (**1**, Scheme S1) was purchased from Santa Cruze Biotechnology. Organic solvents (reagent grade, spectrophotometric grade, and electrochemistry grade) were obtained from Fisher Scientific and Millipore Sigma and used as received. Deuterated solvents were obtained from Cambridge Isotope Laboratories and Millipore Sigma. Tetrabutylammonium hexafluorophosphate, electrochemistry grade, was purchased from Millipore Sigma.

Scheme S1. Synthesis of Aaa.

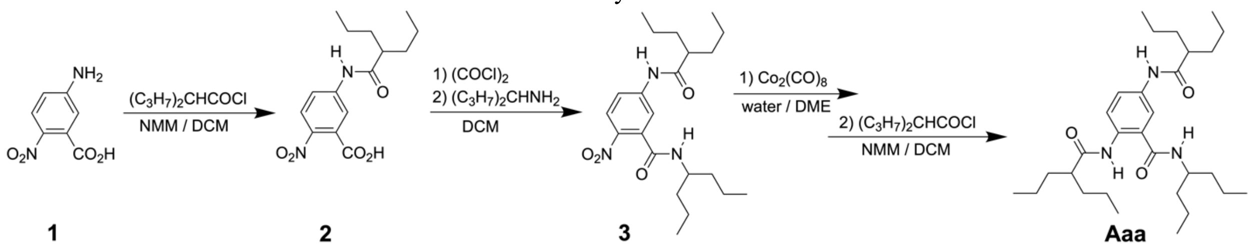

Methods

The ^{1}H NMR spectra were recorded on 600 MHz spectrometers at ambient temperature using $CDCl_3$, CD_3CN, and DMSO-d_6 as solvents. Chemical shifts were confirmed relative to the solvent peaks and verified with previously described results [1]. The intermediates and final compound were also confirmed using high-resolution mass spectrometry (HRMS) and the measured *m/z* ratios were found to agree well with the calculated exact masses.

Synthesis of Aaa (Scheme S1) [1, 2]

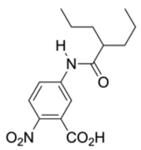

2-nitro-5-(2-propylpentanamido)benzoic acid (2). Under an inert atmosphere, **1** (500 mg, 2.7 mmol) was dissolved in anhydrous dichloromethane (DCM, 5 mL), and the solution was cooled in a dry-ice/acetone bath. Gradually 2,2-di-*n*-propylacetyl chloride (700 µL, 4.1 mmol) was added, followed by addition of *N*-methylmorpholine (1 mL, 9.1 mmol). The mixture was allowed to warm to room temperature and stirred overnight. After diluting it with DCM, the mixture was washed with water and brine, the organic layer was collected, dried over sodium sulfate, and the solvent was removed under reduced pressure. Flash chromatography (silica gell with EtOAc / hexane as a eluent) allowed isolating 457 mg of **2** as an off-white solid (1.48 mmol, 54 % yield). ^{1}H NMR (DMSO-d_6), δ / ppm: 10.60 (s, 1H), 8.03 (m, 2H), 7.87 (d, J = 8.8 Hz, 1H), 2.43 (m, 1H), 1.53 (m, 2H), 1.37 (m, 2H), 1.23 (m, 4H), 0.84 (t, J = 6.7 Hz, 6H); HRMS (ESI) *m/z* calculated for $C_{15}H_{21}N_2O_5^+$ $[M+H]^+$ 309.1445, found 309.1308 $[M+H]^+$**.**

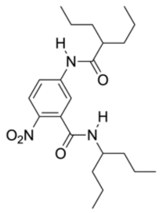

***N*-(heptan-4-yl)-2-nitro-5-(2-propylpentanamido)benzamide (3).** **2** (430 mg, 1.4 mmol) was dissolved in anhydrous DCM under argon. After cooling the solution in a dry-ice/acetone bath, oxalyl chloride (240 µL, 2.8 mmol) was slowly added to it. The reaction was allowed to proceed until completion, verified by adding a drop of the mixture to dry methanol and the formation of the methyl ester of **2**, along with the consumption of **2**, was monitored using TLC. The mixture was dried under reduced pressure and the formed solid residue was redissolved in dry DCM, followed by adding 4-heptylamine (410 µL, 2.8 mmol) and pyridine (225 µL, 2.8 mmol). After stirring overnight at room temperature, the reaction mixture was dissolved in DCM and washed with water, 5% HCl solution and brine, and dried over Na_2SO_4. Flash chromatography (silica gell with EtOAc / hexane as an eluent) allowed isolating 435 mg of **3** as yellow oil (1.07 mmol, 77% yield). ^{1}H NMR (300MHz, $CDCl_3$), δ / ppm: 9.37 (s, 1H), 7.95 (d, J = 9.05 Hz,1H), 7.89 (dd, J = 9.11, 1.98 Hz, 1H), 7.43 (d, J = 2.06 Hz, 1H), 6.07 (d, J = 8.92 Hz, 1H), 4.10 (dtt , J = 7.22, 7.22, 7.15 Hz, 1H), 2.38 (tt, J = 9.03, 5.15 Hz, 1H), 1.64 (m, 12H), 1.37 (m, 4H), 0.97 (t, J = 6.95 Hz, 6H), 0.89 (t, J = 7.18 Hz, 6H); HRMS (ESI) *m/z* calculated for $C_{22}H_{36}N_3O_4^+$ $[M+H]^+$ 406.2628, found 406.2867 $[M+H]^+$.

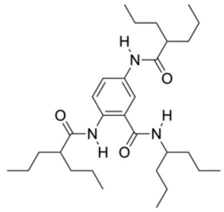

***N,N′*-(2-(heptan-4-ylcarbamoyl)-1,4-phenylene)bis(2-propylpentanamide) (Aaa).** **3** (380 mg, 0.94 mmol) and cobalt octacarbonyl ($Co_2(CO)_8$, 684 mg, 2 mmol) were placed in a pressure tube and dissolved in 5 mL 1,2-dimethoxyethane (DME). After adding a few drops of water (as a proton source), the pressure tube was sealed and heated to 90 °C for one hour. After cooling down, the mixture was filtered, the filtrate was collected, diluted with DCM, washed with water and brine, dried over Na_2SO_4, and the solvent removed under reduced pressure. The obtained residue was redissolved in DCM and cooled on a dry-ice/acetone bath. Gradually 2,2-di-*n*-propylacetyl chloride (400 µL, 2.3 mmol) was added, followed by addition of *N*-methylmorpholine (260 µL, 2.3 mmol). The reaction mixture was allowed to warm up to room temperature and stirred overnight. The mixture was diluted with DCM, washed with aqueous Na_2CO_3 solution and brine, and dried over Na_2SO_4. Flash chromatography (EtOAc / hexane as an eluent) allowed isolating 330 mg of **Aaa** as a white solid (0.65 mmol, 65% yield). ^{1}H NMR (600 MHz, $CDCl_3$) δ / ppm: 10.91 (s, 1H), 8.54 (d, J = 8.9 Hz, 1H), 8.24 (d, J = 2.5 Hz, 1H), 7.13 (s, 1H), 7.11 (dd, J = 8.9, 2.5 Hz, 1H), 5.97 (d, J = 9.3 Hz, 1H), 4.15 (dtt , J = 13.9, 9.1, 5.1 Hz, 1H), 2.28 (tt, J = 9.6, 5.0 Hz, 1H), 2.19 (tt, J = 9.4, 5.1 Hz, 1H), 1.68 (dddd, J = 15.3, 9.2, 7.4, 3.7 Hz, 4H), 1.52 (m, 2H), 1.46 (m, 6H), 1.35 (m, 12H), 0.930 (t , J = 7.4 Hz, 6H), 0.927 (t, J = 7.4 Hz, 6H), 0.898 (t, J = 7.2 Hz, 6H); HRMS (ESI) *m/z* calculated for $C_{30}H_{52}N_3O_3^+$ $[M+H]^+$ 502.4003, found 502.4009. $[M+H]^+$.

Pulse radiolysis

Radiation chemistry is an excellent tool for studying organic radical ions in solvents of high and moderately low polarity. At the initial stage, absorption of the ionizing radiation in diluted solutions affects almost exclusively the solvent. It causes ionization of its molecules. While the radical ions produced are a mixture of free ions and geminate ions, for the molecules investigated here, ion recombination results in disappearance of geminate pairs. Free organic radical cations of solutes can be easily obtained by selecting a suitable solvent.[5] Chlorinated hydrocarbons, such as dichloromethane, are well known to favor the formation of cations of the solute.[6] Pulse radiolysis, therefore, is especially useful for investigating radical ions that have lifetimes that are too short to be observed using spectroelectrochemistry, and yet long enough to be readily detected in the sub-microsecond and microsecond timescales after the initial ionization.

Simplified representation of the reactions proceeding in diluted CH_2Cl_2 solutions of **Aaa** under electron-pulse irradiation are as follow:

$$\mathrm{RCl} \quad \text{–^^^^^→} \ \mathrm{RCl}^{\bullet+} + e^{-} \qquad \text{(S1)}$$

$$\mathrm{RCl} + e^{-} \rightarrow \mathrm{R}^{\bullet} + \mathrm{Cl}^{-} \qquad \text{(S2)}$$

$$\mathrm{RCl}^{\bullet+} + \mathrm{Aaa} \rightarrow \mathrm{RCl} + \mathrm{Aaa}^{\bullet+} \qquad \text{(S3)}$$

Interaction of ionizing radiation with dichloromethane leads to the formation of solvent radical cations, $RCl^{\bullet+}$, and electrons, e^- (eq. S1). Generated electrons, after thermalization, undergo fast dissociative attachment to dichloromethane yielding a relatively unreactive C-centered radicals ($R^\bullet$) and chloride ions, Cl^- (eq. S2). This reaction makes further reductive pathways ineffective. Nevertheless, under the conditions of our experiments, the positive charge from $RCl^{\bullet+}$ can transfer within 100 ns to the solute (eq. S3). Following the timescales of these reactions, therefore, the observed spectra can be ascribed exclusively to $Aaa^{\bullet+}$ (Figure 1c). Meanwhile, $R^\bullet$ absorbs in the deep UV region.

NMR analysis

Assignment of chemical shifts. To assign the chemical shifts to the protons of **Aaa** (Figure 1d,e) we resort to: (1) *J*-coupling patterns in the ^{1}H NMR spectra (Figure 1e); (2) two dimensional (2D) homonuclear NMR spectra, i.e., from correlation spectroscopy (COSY) and nuclear Overhauser effect spectroscopy (NOESY) (Figure S1); and (3) one-dimensional (1D) nuclear Overhauser effect (NOE) spectra (Figure 3 and S2).

The three aromatic protons of 5-substituted anthranilic derivatives form a distinct set of two doublets and one doublet of doublets. The strong coupling between the *ortho*-positioned protons at positions 3 and 4 on the aromatic ring results in 9-Hz splits in their signals. The meta coupling between the protons on positions 4 and 6 causes 2.5-Hz split in their signals. These considerations allow us to assign the doublet with $J = 8.9$ Hz to a_3, the doublet with $J = 2.5$ Hz to a6, and the doublet of doublets with $J_1 = 8.9$ Hz and $J_2 = 2.5$ Hz to a_4 (Figure 1d,e). The COSY of **Aaa** concurs with this assignment, showing only two cross-correlation (off-diagonal) peaks in the aromatic region resulting from the a_3–a_4 and a_4–a_6 coupling (Figure S1a).

Using the thus ascribed chemical shifts to the three aromatic protons, we expand the assignments to the amide protons using through-space interactions, instead of through-bond correlations. The 2D NOESY of **Aaa** shows three cross-correlation peaks in the aromatic and amide region indicative of spatial proximity of these protons (Figure S1b). Specifically, a_4 correlates with a_3 (which is expected since they are on neighboring carbons) and with a slightly broadened singlet which we can ascribe to the nearest amide

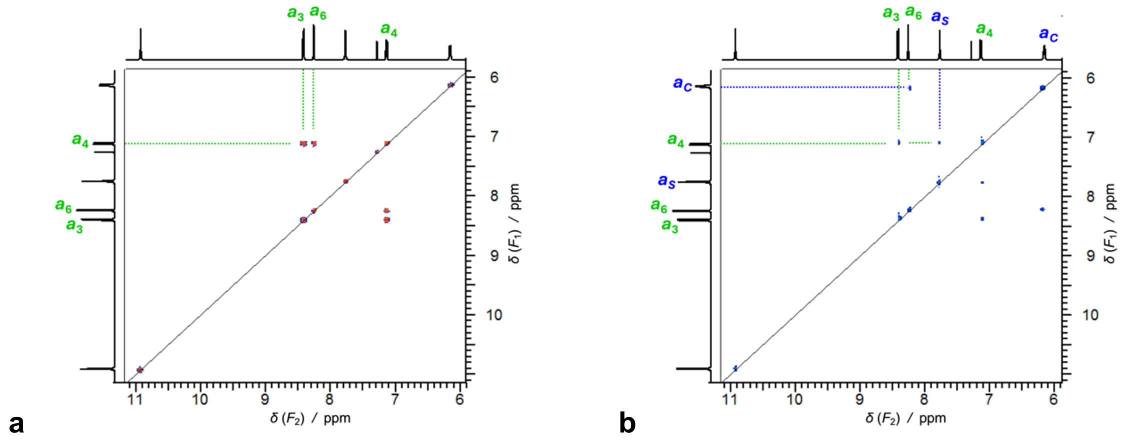

Figure S1. Two-dimensional (2D) ^{1}H NMR spectra of **Aaa**, 50 mM in $CDCl_3$, displaying the aromatic and acidic regions: (a) correlation spectroscopy (COSY), showing homonuclear *J*-coupling; and (b) nuclear Overhauser effect spectroscopy (NOESY), showing through-space coupling.

proton, a_S (Figure S1b, 1d and 3d). Concurrently, a_6 correlates through space only with a broad doublet, which ought to be the proton of the *C*-terminal amide, a_C (Figure S1b, 1d and 3d).

To expand the assignment to the other protons, we resort to 1D NOE spectroscopy, which also allows us to lower the sample concentration. The protons attached to the tertiary carbons of the 4-heptyls capping the amides show distinct well-separated multiplets between 2 and 4.5 ppm (Figure 1e). Saturation of the a_S proton causes an enhancement of the most upfield shifted signal of these multiplets, i.e., at 2.19 ppm, which we assign to h_S since it is the closest aliphatic proton to the side-chain amide (Figure 3a,d). Indeed, saturation of the thus assigned h_S multiplet enhances the a_S signal (Figure S2a). Saturating the most downfield-shifted of these multiplets, i.e., at 4.15 ppm, increases the signal of the C-terminal amide proton, a_C (Figure S2). It allows us to assign this multiple to the h_C proton that is attached to the tertiary carbon of the chain capping the *C*-terminal amide (Figure S2a).

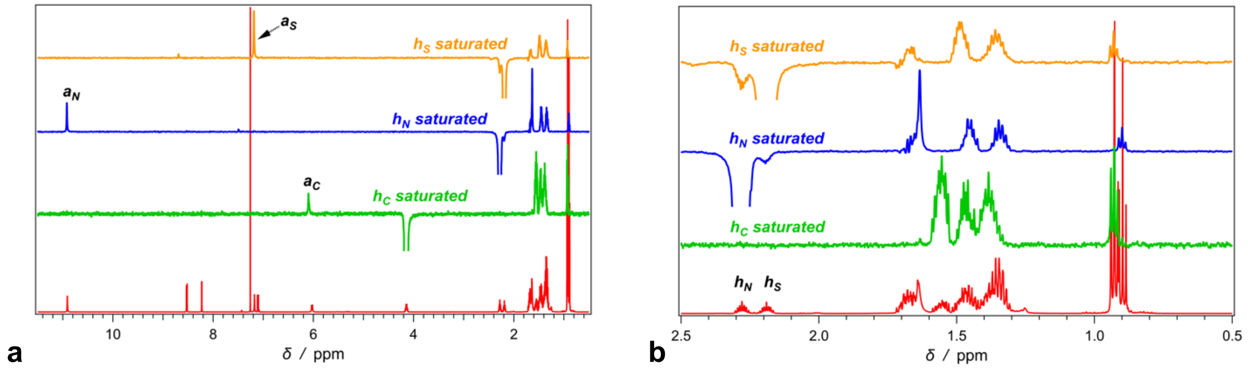

Figure S2. One dimensional (1D) nuclear Overhauser effect (NOE) spectra of **Aaa**, 5 mM in $CDCl_3$, obtained from saturating the signals of the protons on the tertiary carbons, h_C, h_N, and h_S, of the branched aliphatic chains capping the three amides (Figure 1d). The corresponding ^{1}H NMR spectrum is shown in red on the bottom of the graphs. (a) The 1D NOE and ^{1}H NMR spectra; and (b) the aliphatic region of these spectra.

Saturating the third of these multiplets, i.e., at 2.28 ppm, shows distinct enhancement of the most downfield signal at around 11 ppm (Figure S2a). Therefore, we assign the 2.28-ppm multiplet to h_N and the 11-ppm singlet to a_N (Figure 1e). The NOE excitation of the three heptyl protons attached to tertiary carbons produces signal enhancements that allows detailed assignment of the NMR peaks in the aliphatic region (Figure S2b). Even of the overlapping triplets of the methyls at the ends of the alkyl chains are clearly revealed on the 1D NOE spectra (Figure S2b).

Concentration dependence of the chemical shifts. Solutions of **Aaa** in $CDCl_3$ with concentrations between 1 and 60 mM were prepared and their ^{1}H NMR spectra were recorded (Figure S3a). An increase in sample concentration affects the chemical shift of the side-chain amide proton, a_S (Figure 1d), the most. The downfield shift of the a_S signal amounts to about 0.5 ppm as the **Aaa** concentration increases to 60 mM (Figure S3b). The signal from the other amide proton that is not hydrogen bonded, i.e., a_C (Figure 1d), also undergoes a downfield shift with the increase in concentration (Figure S3b). Conversely, the chemical shift of the hydrogen-bonded amide proton, a_N (Figure 1d), is invariant to concentration changes. The signal from the aromatic proton at the third position, a_3 (Figure 1d), undergoes an upfield shift (Figure S3b), which is consistent with the formation of aggregates where this proton is situated over an aromatic ring inducing a shielding effect.[7] Concurrently, the deshielding of a_S and a_C can originate from hydrogen bonding of these two protons upon aggregation of **Aaa**.

Temperature dependence. To minimize aggregation at room temperature, we prepare **Aaa** solutions in deuterated solvents with concertations that do not exceed 10 mM. That is, the a_S peak of **Aaa** in $CDCl_3$ is shifted upfield in comparison with the signal from $CHCl_3$ (Figure 3a). Starting from 25 °C, we lowered the temperature of the NMR probe and recorded a spectrum at every five degrees. For acetonitrile, we limited the lowest temperature to -40 °C because the melting point of CD_3CN is -46 °C. While we did not add internal temperature probes to the samples, calibration of the probe with such probes revealed up to two-degree-C deviations for the investigated temperature range.

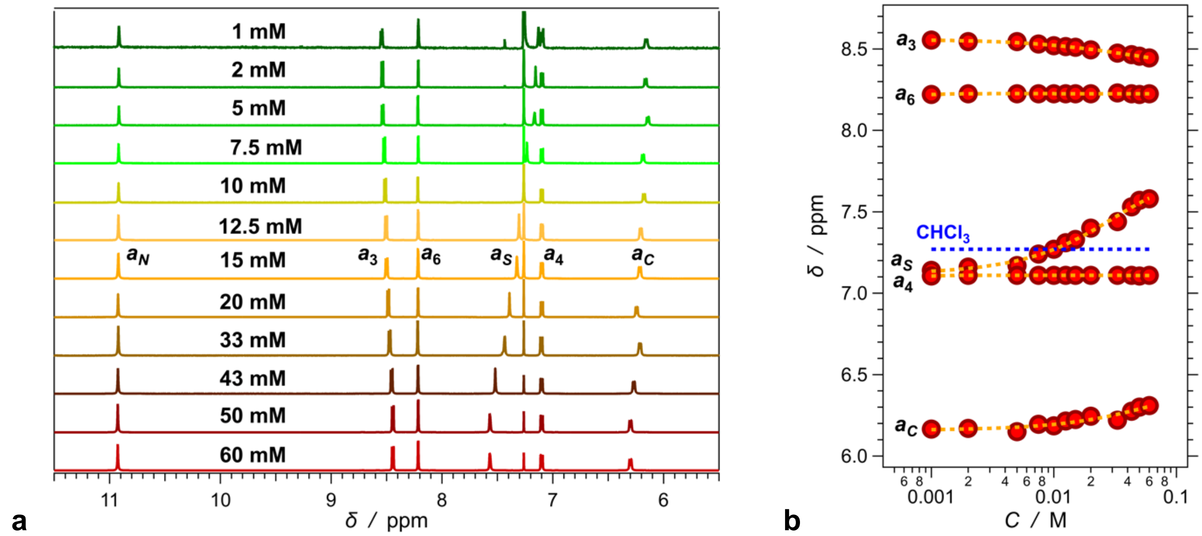

Figure S3. Concentration dependence of the ^{1}H NMR spectra of **Aaa** in $CDCl_3$, focusing on the aromatic and amide protons. (a) Spectra recorded for concentrations varying between 1 and 60 mM. (b) Dependence of the chemical shifts of the aromatic protons and non-hydrogen bonded amide protons, a_S and a_N (Figure 1d), on the concentration of **Aaa**.

Computational studies

Table S1. Electronic energies of the *E* conformer and two transition states relative to the *Z* conformer. Data calculated at the CAM-B3LYP-D3(BJ)/6-311+G(d,p) level of theory.

Environment	ε_r	TS_1	*E* conformer	TS_2
Vac	1	18.03	12.11	20.21
Tol	2.4	14.27	9.11	16.21
$CHCl_3$	4.9	11.75	6.65	13.28
CH_2Cl_2	8.9	10.13	4.88	11.04
CH_3CN	36.6	8.45	2.94	8.16
DMSO	46.7	8.29	2.76	8.19
Water	78.4	8.09	2.52	7.70

Table S2. Enthalpies at 298 K of the *E* conformer and two transition states relative to the *Z* conformer. Data calculated at the CAM-B3LYP-D3(BJ)/6-311+G(d,p) and rigid rotor – harmonic oscillator levels of theory.

Environment	ε_r	TS_1	*E* conformer	TS_2
Vac	1	15.70	12.37	17.86
Tol	2.4	14.66	9.07	8.27
$CHCl_3$	4.9	11.36	6.64	6.89
CH_2Cl_2	8.9	8.35	4.89	9.26
CH_3CN	36.6	6.66	3.12	5.18
DMSO	46.7	6.48	2.95	6.36
Water	78.4	6.46	2.71	5.38

Table S3. Gibbs energies at 298 K and 100 kPa of the *E* conformer and two transition states relative to the *Z* conformer. Data calculated at the CAM-B3LYP-D3(BJ)/6-311+G(d,p) and rigid rotor – harmonic oscillator levels of theory.

Environment	ε_r	TS_1	*E* conformer	TS_2
Vac	1	23.34	11.00	25.50
Tol	2.4	18.73	10.14	14.45
$CHCl_3$	4.9	17.95	7.25	13.00
CH_2Cl_2	8.9	13.05	5.36	15.12
CH_3CN	36.6	12.82	2.22	11.01
DMSO	46.7	11.32	1.95	10.76
Water	78.4	8.90	1.74	9.63

Table S4. Some interatomic distances, *d*, corresponding to non-covalent contacts of **Aaa** in DCM illustrating the steric hindrance responsible for twisting the amides out of the plane of the aromatic ring (Chart 1).

**Aaa$^{(Z)}$**		**Aaa$^{(E)}$**	
atoms	d / Å	atoms	d / Å
$a_N \cdots O$	1.85 [a]	$a_N \cdots O$	1.88 [a]
$a_6 \cdots O$	2.18	$a_4 \cdots O$	2.20
$a_3 \cdots O$	2.21	$a_3 \cdots O$	2.23
$a_6 \cdots a_C$	2.07	$a_6 \cdots a_C$	2.17
$a_4 \cdots a_S$	2.28	$a_6 \cdots a_S$	2.24

[a] Hydrogen bonding between the amides at positions 1 and 2 (Chart 1).

Table S5. Twisting of the *C*-terminal amide, at position 1 (Chart 1), out of the plane of the aromatic ring as illustrated by the dihedral angle ψ_1 of C_6-C_1-C(O)-N(H); and of the *N*-terminal amide at position 2 (Chart 1), out of the plane of the aromatic ring as illustrated by the dihedral angle ψ_2 of C_3-C_2-N(H)-C(O).

solvent	ψ_1 / deg		ψ_2 / deg	
	**Aaa**$^{(Z)}$	**Aaa**$^{(E)}$	**Aaa**$^{(Z)}$	**Aaa**$^{(E)}$
Vac	22.3	29.0	−9.2	−12.7
Tol	25.5	31.3	−12.5	−16.3
$CHCl_3$	28.1	32.8	−16.2	−19.3
CH_2Cl_2	29.6	33.5	−18.1	−20.5
CH_3CN	31.0	34.2	−19.8	−21.7
DMSO	31.1	34.3	−20.0	−21.9
water	31.3	34.4	−20.2	−22.1

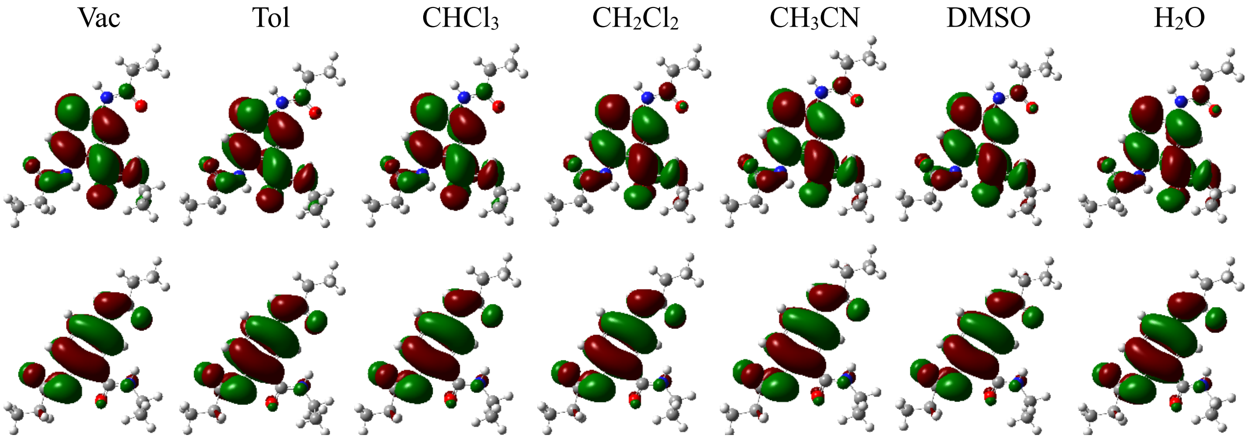

Figure S4. Shapes of the frontier orbitals of the Z conformer of **Aaa** in various solvation environments. Top row – LUMO orbitals, bottom row – HOMO orbitals.

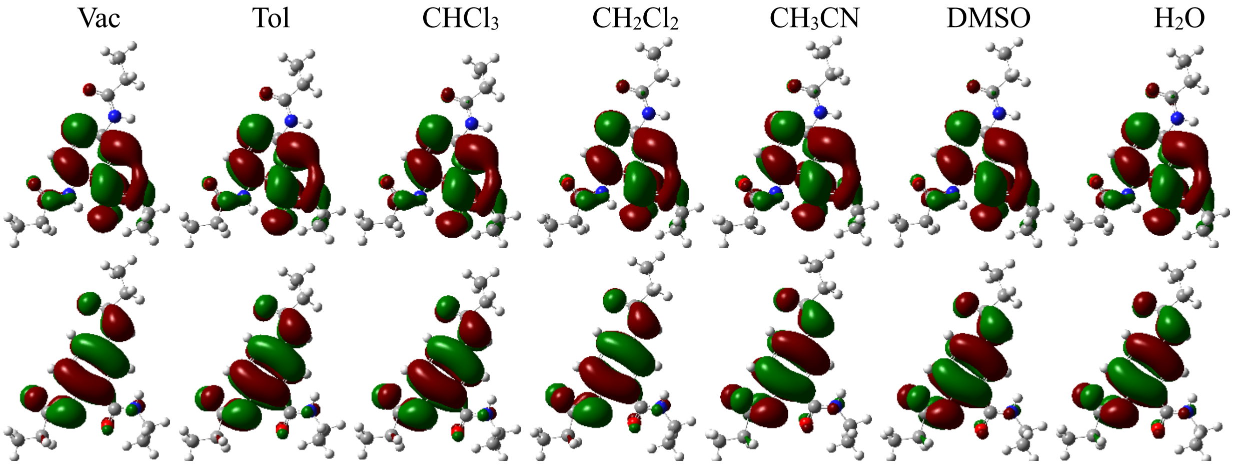

Figure S5. Shapes of the frontier orbitals of the E conformer of **Aaa** in various solvation environments. Top row – LUMO orbitals, bottom row – HOMO orbitals.

Bond ellipticity. To evaluate the π-conjugation between the three amides and the aromatic ring of **Aaa**, we resort to estimation the ellipticity of these bonds. The ellipticity of bonds between the amides and the aromatic ring is indicative of π-electron delocalization (π-conjugation).[8, 9] An elevated degree of ellipticity typically signifies enhanced conjugation, which stabilizes the molecule in planar conformations through electronic effects, thereby influencing the overall molecular geometry. The N-terminal and side-chain amides exhibit stronger π-conjugation with the aromatic ring compared to the C-terminal amide, suggesting that these amides are more electronically stabilized, maintaining a flatter orientation relative to the aromatic ring. Despite the presence of hydrogen bonding, which typically favors planarity, the C-terminal amide remains twisted at a significantly larger angle compared to the N-terminal amide. The electronic stabilization due to π-conjugation predominates over steric hindrance in dictating preferred conformations. Even in transition states, partial π-conjugation between the side-chain amide and the aromatic ring persists, reinforcing the significance of these electronic interactions across various molecular states. This analysis reveals an interplay between electronic effects, molecular geometry, and hydrogen bonding in shaping the structure and stability of **Aaa**, illustrating the essential understanding of molecular dynamics and stability.

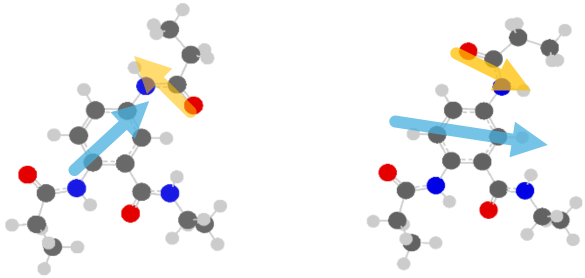

Z ***E***

Figure S6. DFT-optimized structures of the *Z* and *E* conformers of **Aaa** along with their dipoles (blue arrows) and the local amide dipole (orange arrow) at position C_5. The *E* conformer enjoys a higher $|\mu| \approx 8$ D due to the dipole dis-alignment between the backbone and the amide. The *Z* conformer main dipole is rotated to an almost orthogonal angle of 87 degrees to the above-mentioned amide, thus the $|\mu| \approx 4$ D.

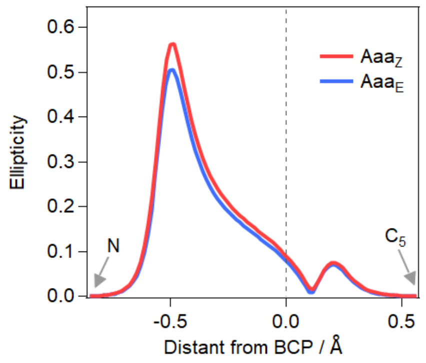

Figure S7. Ellipticity of the bond between the side-chain amide and the aromatic ring, i.e., C_5-N, illustrating the extent of their π characters, as described previously.[8, 9] The *Z* conformer is able to retain more of its electron density closer to the amide in part due to the orientation of its amide dipole pulling away from the **Aaa** back bone, whereas the *E* conformer donates more of its electron density than the *Z* from the amide at position 5 to the **Aaa** back bone. The ellipticity also shows that the *Z* conformer has a higher propensity of being stabilized than the *E* conformer.

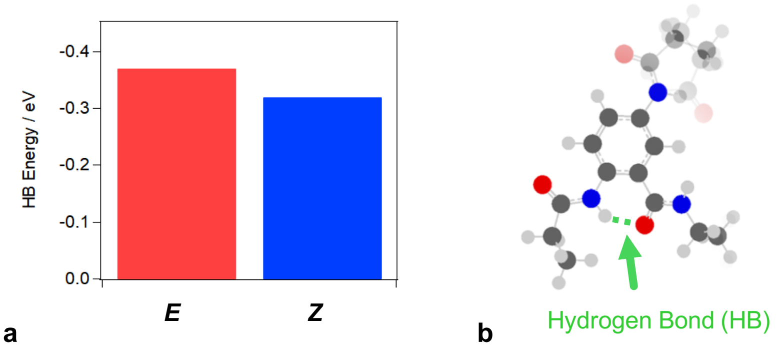

Figure S8. Hydrogen Bond strength differences between the *Z* and *E* conformers.[8, 10] The *E* conformer has a higher bond strength by about 0.06 eV than the *Z* conformer due in part to the capability of the amide at position C_5 to donate a larger portion of its electron density to the backbone of the **Aaa** than in the *Z* conformer.

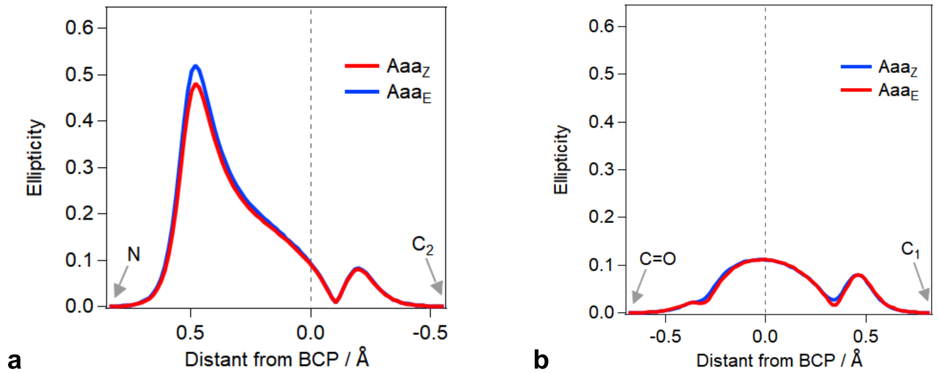

Figure S9. Ellipticity of the bonds of the aromatic ring with (a) the *N*-terminal amide, i.e., C_2-N, and (b) the *C*-terminal amide, i.e., C_1-C, illustrating the extent of their π characters.[8, 9] The *Z* conformer has less of an electron density along its bond in contrast to its *E* counterpart. The electron density from the amide at position 5 donates almost all of its excess spelled electron density to the amide at position 2. Change of conformations has no effect to on the C terminus.

Natural bonding orbital (NBO) analysis confirms that the hybridization of N-C bonding orbitals located at nitrogen atoms at the *N*-terminus and the side chain is very close to sp^2 with the exact percentage of the *p*-character varying between 62% and 66% for individual solvation environments. This finding agrees with a strong delocalization of the lone pair within both the aromatic system and carbonyl moiety. Interestingly, the *p*-character of the N-C bonding orbital toward the aromatic ring is always higher than for the bonding orbital toward the carbonyl, as listed in Table S6. Analogous N-C bonding orbital towards the aliphatic side chain at the C-terminus has the highest *p*-character among the analyzed orbitals centered on nitrogen atoms, confirming the weakest delocalization of the lone pair and thus a prevailing sp^3 hybridization in that direction. There are negligible differences predicted by the NBO between both conformers and various solvents.

Table S6. Results of the natural bond orbital analysis: fraction of the *p*-character of the orbitals centered at nitrogen atoms that participate at formation of all nitrogen-carbon bonds in **Aaa** molecule, or at the carbonyl carbon of the *C*-terminus. Carbon atoms adjacent to nitrogens are labeled to distinguish aromatic (C_A), carbonyl (C_O), and aliphatic C_C carbon atoms.

Conformer			**Aaa$^{(Z)}$**				
Moiety	*N*-terminus		Side chain		*C*-terminus		
Bond	N-C_A	N-C_O	N-C_A	N-C_O	N-C_C	N-C_O	C_O-C_A
Gas	0.6461	0.6347	0.6313	0.6295	0.6535	0.6283	0.6334
Toluene	0.6463	0.6336	0.6322	0.6294	0.6534	0.6271	0.6350
Chloroform	0.6465	0.6332	0.6326	0.6292	0.6536	0.6266	0.6358
Dichloromethane	0.6465	0.6329	0.6327	0.6291	0.6536	0.6263	0.6364
Acetonitrile	0.6467	0.6328	0.6328	0.6290	0.6537	0.6259	0.6369
Dimethylsulfoxide	0.6467	0.6327	0.6328	0.6290	0.6537	0.6259	0.6369
Water	0.6467	0.6327	0.6328	0.6290	0.6537	0.6258	0.6370
Conformer			**Aaa$^{(E)}$**				
Moiety	*N*-terminus		Side chain		*C*-terminus		
Bond	N-C_A	N-C_O	N-C_A	N-C_O	N-C_C	N-C_O	C_O-C_A
Gas	0.6446	0.6347	0.6297	0.6293	0.6534	0.6295	0.6324
Toluene	0.6450	0.6338	0.6311	0.6290	0.6534	0.6278	0.6350
Chloroform	0.6455	0.6334	0.6317	0.6290	0.6535	0.6269	0.6365
Dichloromethane	0.6457	0.6332	0.6321	0.6289	0.6535	0.6263	0.6374
Acetonitrile	0.6460	0.6328	0.6324	0.6289	0.6537	0.6259	0.6382
Dimethylsulfoxide	0.6460	0.6328	0.6324	0.6289	0.6537	0.6258	0.6383
Water	0.6460	0.6328	0.6324	0.6290	0.6537	0.6258	0.6384

Second-order perturbative energies associated with hyper-conjugation donor-acceptor relationships of orbitals reveals that there are very strong stabilizing interactions between the lone-pair orbitals at both *N*-terminus and side chain nitrogen atoms with the carbonyl antibonding orbital and with the closest aromatic carbon-carbon antibonding orbital. These hyper-conjugation interactions are always stronger with the carbonyl moiety than with the aromatic system, as listed in Table S7. This conjugation with the aromatic core is stronger at the *N*-terminus (where it decreases as polarity rises) than at the side chain for both conformers (where it increases as polarity rises). Interestingly, the conjugation of the lone-pair orbital of the side-chain nitrogen with the adjacent carbonyl is significantly weaker for the **Aaa$^{(E)}$** conformer in low-polarity media. Obviously, the conjugation of the *C*-terminal nitrogen lone pair is possible only with the carbonyl, and not with the aromatic core. Still, there is a non-negligible hyperconjugation of this lone pair with the aliphatic moiety at the *C*-terminus. Table S8 reveals that there are non-negligible hyperconjugation effects where the lone-pair orbitals from the *C*-terminus oxygen atom act as donors towards the antibonding (N−H)* orbitals. It agrees with a formation of a strong hydrogen bond at that site. Based on the second-order perturbation energies, this effect is stronger for the **Aaa$^{(Z)}$** conformer, and it naturally wanes as the polarity increases.

Table S7. Results of the second order perturbation theory analysis of Fock matrix in the natural bond orbital basis: second-order energies due to hyper-conjugation effects in eV. Data are listed for important interactions of donor lone-pair orbitals centered at nitrogen atoms in **Aaa** molecule. Acceptor antibonding orbitals are located near aromatic (C_A), carbonyl (C_O), and aliphatic C_C carbon atoms.

Conformer	**Aaa$^{(Z)}$**					
Moiety	*N*-terminus		Side chain		*C*-terminus	
Donor	N_{LP}		N_{LP}		N_{LP}	
Acceptor antibonding	$(C_A\text{-}C_A)^*$	$(C_O\text{=}O)^*$	$(C_A\text{-}C_A)^*$	$(C_O\text{=}O)^*$	$(C_O\text{-}C_C)^*$	$(C_O\text{=}O)^*$
Gas	1.979	3.097	1.527	3.151	0.313	3.468
Toluene	1.898	3.196	1.576	3.221	0.305	3.551
Chloroform	1.826	3.254	1.618	3.267	0.302	3.599
Dichloromethane	1.785	3.287	1.657	3.295	0.300	3.621
Acetonitrile	1.745	3.320	1.694	3.307	0.297	3.643
Dimethylsulfoxide	1.742	3.322	1.697	3.309	0.297	3.644
Water	1.737	3.326	1.701	3.313	0.297	3.646
Conformer	**Aaa$^{(E)}$**					
Moiety	*N*-terminus		Side chain		*C*-terminus	
Donor	N_{LP}		N_{LP}		N_{LP}	
Acceptor antibonding	$(C_A\text{-}C_A)^*$	$(C_O\text{=}O)^*$	$(C_A\text{-}C_A)^*$	$(C_O\text{=}O)^*$	$(C_O\text{-}C_C)^*$	$(C_O\text{=}O)^*$
Gas	1.891	3.094	1.540	2.634	0.317	2.972
Toluene	1.805	3.215	1.561	2.615	0.309	3.192
Chloroform	1.714	3.287	1.638	2.642	0.304	3.339
Dichloromethane	1.691	3.330	1.660	2.670	0.301	3.436
Acetonitrile	1.670	3.468	1.680	3.339	0.298	3.555
Dimethylsulfoxide	1.667	3.472	1.681	3.345	0.298	3.563
Water	1.664	3.479	1.680	3.352	0.298	3.575

Table S8. Results of the second order perturbation theory analysis of Fock matrix in the natural bond orbital basis: second-order energies due to hyper-conjugation effects in eV. Data are listed for important interactions of donor lone-pair orbitals centered at carbonyl oxygen atoms in Aaa molecule that participate at intramolecular hydrogen bonding. Acceptor antibonding orbitals are located near the N-H moiety forming that hydrogen bond.

Conformer	**Aaa$^{(Z)}$**	**Aaa$^{(E)}$**
Donor	O_{LP}	
Acceptor antibonding	(N-H)*	
Gas	0.455	0.326
Toluene	0.408	0.301
Chloroform	0.367	0.280
Dichloromethane	0.341	0.271
Acetonitrile	0.317	0.261
Dimethylsulfoxide	0.315	0.261
Water	0.313	0.259

Evaluation of the hydrogen bonding. Intramolecular non-covalent interactions (NCI) were analyzed in the framework of the quantum theory of atoms in molecules (QTAIM). Important sites of such NCI were identified with respect to occurrence of critical points in the plot of the reduced density gradient (RDG) versus the sign of the second density Hessian eigenvalue (λ_2).[11] The data handling required for this NCI analysis was completed using the MultiWFN package.[12] Natural bond orbital analysis was performed to

analyze the hybridization of the nitrogen atoms within the **Aaa** molecule.[13] Hyperconjugation effects of the lone-pair orbitals at nitrogen atoms in **Aaa** were analyzed using the second order perturbation theory analysis of Fock matrix in the natural bond orbital basis.[14]

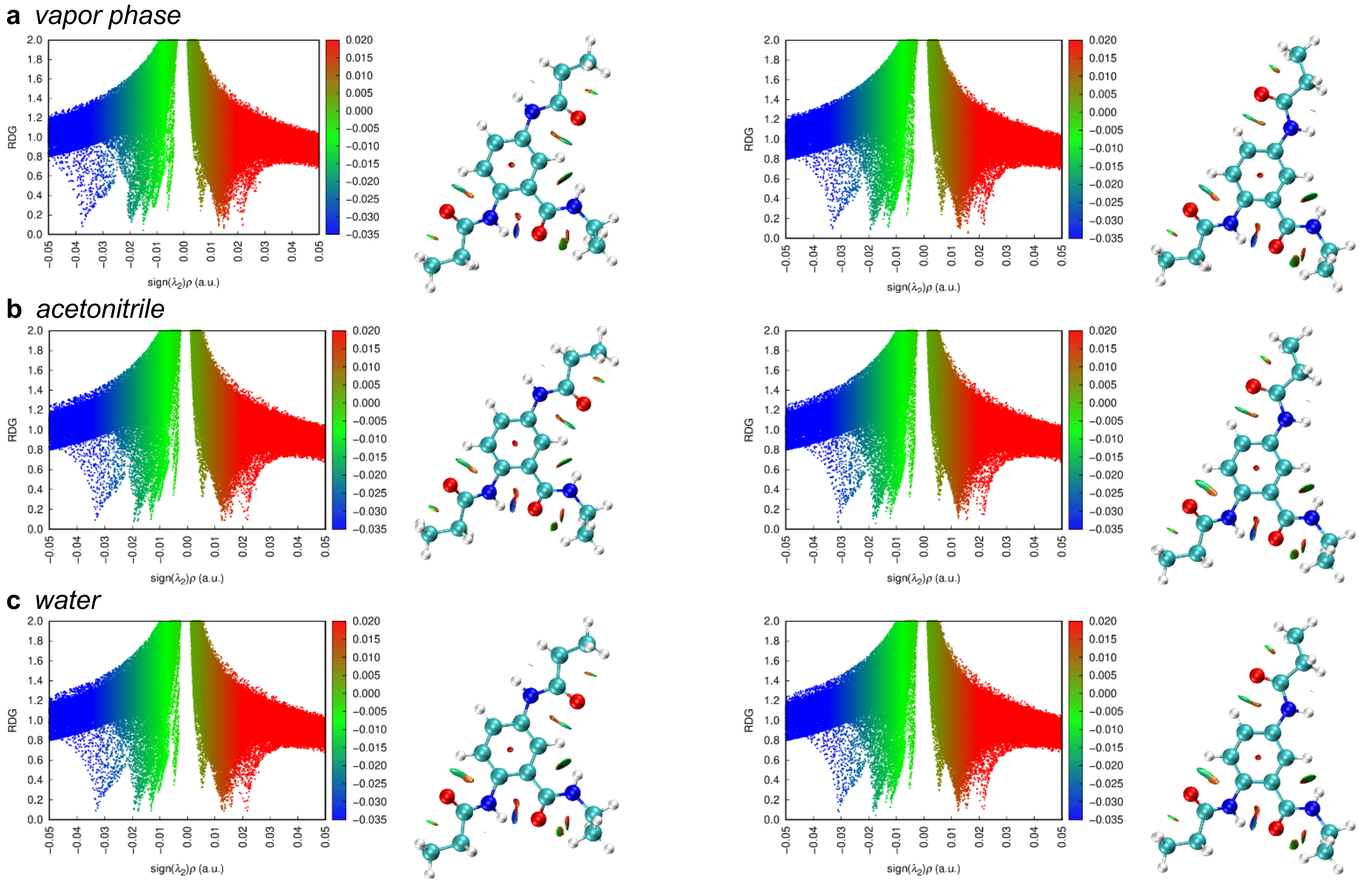

Figure S10. Analysis of intramolecular non-covalent interactions in **Aaa$^{(Z)}$** (left column) and **Aaa$^{(E)}$** (right column) conformers in selected solvation environments. Data presented in terms of the reduced density gradient and the sign of the second density Hessian eigenvalue (λ_2), all values given in atomic units. Figure 2e and f show the results for chloroform.

The NCI analysis confirms that the only site where a strong intramolecular hydrogen bond can form is between the N-terminal N-H moiety and C-terminal C=O moiety of both **Aaa$^{(Z)}$** and **Aaa$^{(E)}$** conformers. This finding is revealed by the blue isosurfaces in that molecular region (Figure S10 and 2e,f). A blue spike of the attractive region of the reduced density plot in the interval of sign(λ_2)ρ value from −0.4 a.u. to −0.3 a.u. in Figure S10 and 2e,f corresponds to this hydrogen bonding. Remaining spikes in the region 0.0 a.u. > sign(λ_2)ρ > −0.2 a.u. are attributed to weaker dispersion attractive interactions of the remaining carbonyl oxygen atoms to proximate hydrogen atoms (green isosurfaces in Figure S10 and 2e,f). Still, these interactions are closely coupled with repulsions from adjacent non-hydrogen atoms (orange and red isosurfaces in Figure S10 and 2e,f). A detailed comparison of the NCI analysis results does reveal that the intramolecular hydrogen bond is somewhat stronger in the vapor in **Aaa$^{(Z)}$** than in **Aaa$^{(E)}$** as the leftmost critical RDG point (i. e. RDG spike approaches zero value) is located at more negative sign(λ_2)ρ values for **Aaa$^{(Z)}$**. Importantly, the precise position of this critical point shifts appreciably to larger sign(λ_2)ρ values upon solvation of **Aaa$^{(Z)}$** in polar media (see Figure and 2e and the left column of Figure S10). This indicates

some waning of this intramolecular hydrogen bond for **Aaa$^{(Z)}$** due to solvation. Notably, this trend is also present for **Aaa$^{(E)}$**, but appreciably less significant. There are no important differences in positions of other critical RDG points that correspond to remaining intramolecular van der Waals attractions or steric repulsions.

Table S9. ^{1}H NMR chemical shifts of the amide and aromatic protons (Figure 1d) of the *Z* and *E* conformers of **Aaa** calculated at the B3LYP/6-311+(2d,p) level of theory with the GIAO method for static optimized geometries of both monomer conformers.

Conformer	**Aaa$^{(Z)}$**					
Moiety	*N*-terminus		Side chain		*C*-terminus	
Bond	a_N	a_3	a_S	a_4	a_C	a_6
Gas	12.0694	9.5823	6.4150	6.7576	6.1828	9.3369
Toluene	11.8412	9.3744	6.7388	6.9091	6.2466	9.2985
Chloroform	11.6235	9.1965	6.9254	6.9254	6.2904	9.2930
Dichloromethane	11.4729	9.0956	7.0390	7.0433	6.3020	9.3152
Acetonitrile	11.3351	8.9982	7.1483	7.0917	6.3148	9.3350
Dimethylsulfoxide	11.3221	8.9890	7.1577	7.0959	6.3162	9.3358
Water	11.3057	8.9769	7.1698	7.1015	6.3180	9.3367
Conformer	**Aaa$^{(E)}$**					
Moiety	*N*-terminus		Side chain		*C*-terminus	
Bond	H_N…O	H_A…O	H_N…H_A	H_A…O	H_N…H_A	H_A…H_N
Gas	11.1317	9.5070	6.2275	9.3575	5.2786	6.5926
Toluene	11.0405	9.2665	6.6034	9.2878	5.6027	6.7804
Chloroform	10.9561	9.0974	6.8224	9.2349	5.8120	6.9018
Dichloromethane	10.9224	9.0080	6.9590	9.1970	5.9460	6.9835
Acetonitrile	10.8973	8.9170	7.0884	9.1303	6.0885	7.0706
Dimethylsulfoxide	10.8938	8.9085	7.0994	9.1204	6.1017	7.0786
Water	10.8896	8.8977	7.1139	9.1060	6.1185	7.0891

Density functional theory and the Gauge-Independent Atomic Orbital (GIAO) [15] were used to model ^{1}H NMR chemical shifts of the relevant hydrogen atoms of the *E* and *Z* conformers of **Aaa** that are affected by the mutual orientation of the aromatic core and the side amide chains. These calculations were carried out at the B3LYP/6-311+G(2d,p) level of theory in Gaussian and listed in Table S9. Comparison of the DFT-obtained results with experimentally recorded spectra reveals that the computational estimates do not really represent the measured chemical shifts (Figure S11). Indeed, the computations are only for the two optimized conformers and do not accout for the whole ensembles of structures in the solutions. For the *N*-terminal amide proton, a_N, for example, the computed chemical shift for both conformers overestimate the deshielding of this proton with the result for **Aaa$^{(E)}$** closer than that for **Aaa$^{(Z)}$** conformer. Conversely, for the *C*-terminal amide proton, a_C, both computed results for CD_3CN underestimate the deshielding with the values for the *Z* conformer closer to the experimental measurements than that for the *E* conformer (Figure 9). Furthermore, the experimental spectra show that solvent polarity deistically affects the chemical shifts in the aromatic spectral region, while the computed results for the DFT structures show less than 0.3-ppm shifts when transitioning between $DCCl_3$ and CD_3CN (Figure S11, Table S9). These findings preclude drawing of any reliable conclusions based on comparison of the theoretical with the experimental results.

Nevertheless, results listed in Table S9 indicate that the amide hydrogen atom at the *N*-terminus experiences the strongest shielding due to its participation at the strong intramolecular hydrogen bond. Although this hydrogen atom exhibits the largest chemical shifts for both conformers in all considered solvation models, it is important to analyze the variation of its chemical shift due to the changes of solvent polarity. Switching from the vapor to water solution, the intramolecular hydrogen bond becomes somewhat less important as this particular chemical shift drops by 0.76, whereas the same drop amounts to only 0.24 ppm for **Aaa$^{(E)}$**.

This difference supports our findings from the NCI analysis and confirms that the intramolecular hydrogen bond wanes more significantly in **Aaa(Z)** upon solvation than in **Aaa(E)**, which possibly contributes to the relative stabilization of **Aaa(E)** in more polar environments. Even in water, this chemical shift is predicted to be still slightly larger in **Aaa(Z)** though.

On the other hand, **Aaa(E)** conformer exhibits more significant increase of the shielding for a_S and a_C amidic hydrogen signals as the solvent polarity rises. Still, both a_S and a_C signals are predicted to occur at lower chemical shifts in **Aaa(E)** than in **Aaa(Z)**, which holds for all solvation environments. This is in agreement with the observed temperature-induced decrease of the experimental a_S and a_C chemical shifts that are depicted in Figure 3, although the experimentally observed variation is more important than what would correspond to the calculated differences between a_S and a_C signals for **Aaa(E)** and **Aaa(Z)**. Due to the relative computational simplicity of the NMR model, however, quantitative accuracy of the predicted chemical shifts in not the primary goal in this work. Such calculated data serve to interpret the observed phenomena instead. Otherwise, there are obvious differences between the chemical shift signals a_4 and a_6 of both conformers. Due to the swap of the side-chain amide plain, the hydrogen atom being closer to the amide oxygen experiences larger shielding (a_6 in **Aaa(Z)** and a_4 in **Aaa(E)**).

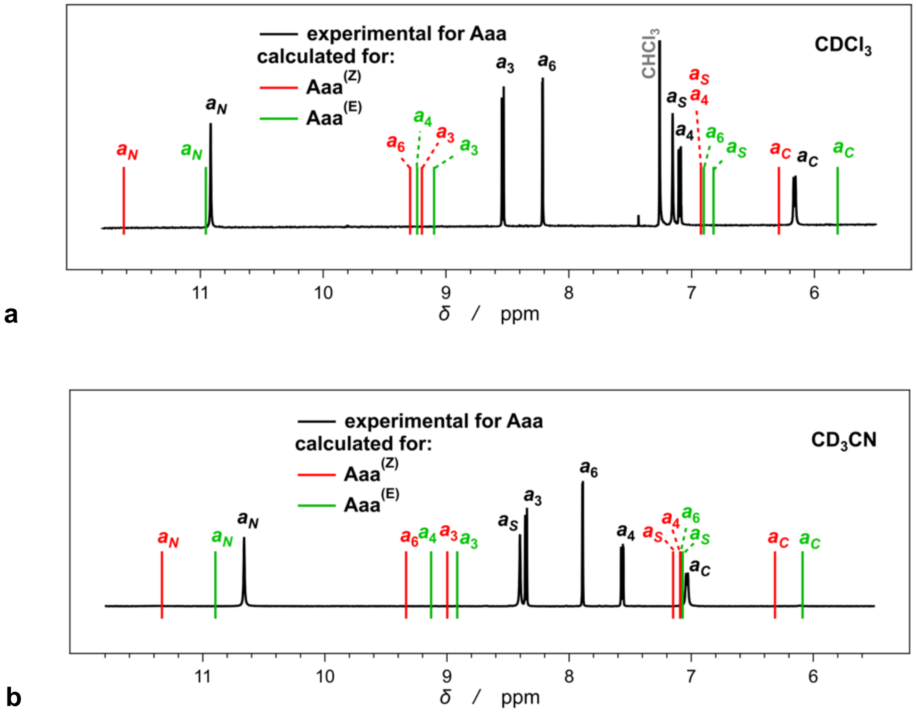

Figure S11. Comparison of the experimentally measured NMR spectra of **Aaa** in (a) $CDCl_3$ (Figure 1e) and (b) CD_3CN (20 °C, Figure 3b) with the computed chemical shifts of the aromatic and amide protons of the *E* and *Z* conformers (Table S9).

Energy minima and transition states. Following the relaxed scan along the pathway that varies the key dihedral angle, covering the whole 360 degrees, transition state geometries were reoptimized with the QST3 algorithm. The minimum-energy conformers indeed exhibit only positive vibrational frequencies, while the transition-state structures show only a single imaginary frequency (Figures S10 and S11).

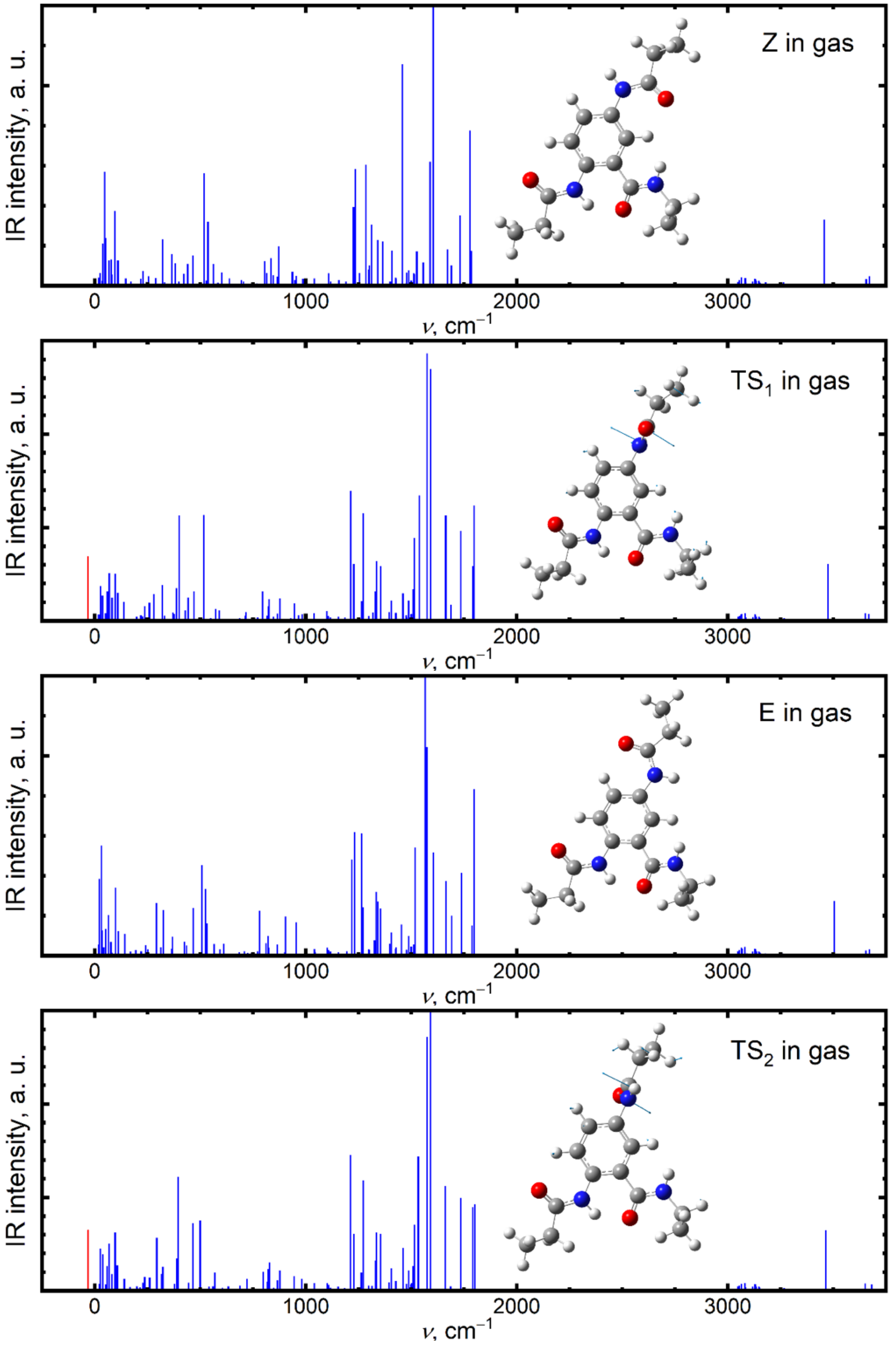

Figure S12. Vibrational frequencies calculated at the CAM-B3LYP-D3(BJ)/6-311+G(d,p) level of theory for the two stable gas phase **Aaa**$^{(Z)}$ and **Aaa**$^{(E)}$ conformers (with all frequencies real) and for the two transition states separating those (both with a single imaginary mode the eigenvectors of which are depicted).

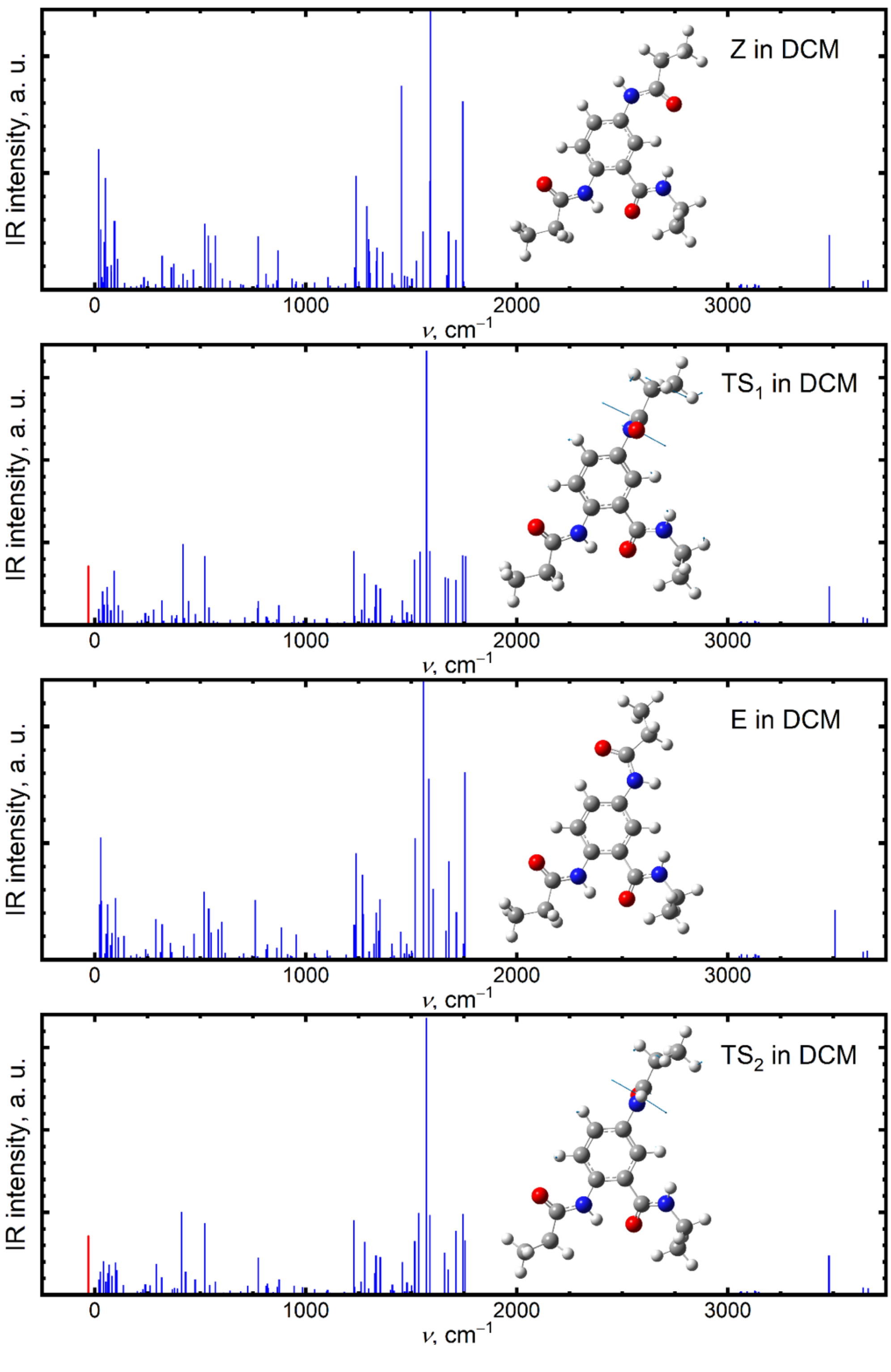

Figure S13. Vibrational frequencies calculated at the CAM-B3LYP-D3(BJ)/6-311+G(d,p) level of theory for the two stable **Aaa$^{(Z)}$** and **Aaa$^{(E)}$** conformers (with all frequencies real) solvated in dichloromethane and for the two transition states separating those (both with a single imaginary mode the eigenvectors of which are depicted).

Transition-state energies. Employing a range-separated hybrid DFT functional, which sits just below the top of the Jacobbs' ladder in the hierarchy of DFT functionals, minimizes the delocalization error nearly as much as possible within the DFT framework [16].

To refine the relative electronic energies of both **Aaa** conformers and of the transition states that separate them on the potential energy hypersurface, single-point *ab initio* energy calculations were performed for the CAM-B3LYP-D3 optimized molecular structures. Domain-based local pair natural orbital formulation of the coupled clusters theory, DLPNO-CCSD(T),[17] and automated generation of auxiliary basis sets,[18] as implemented in Orca software[19] were used for this purpose. Tight criteria TightPNO within the DLPNO formalism, the iterative treatment of the triple excitations[20] and an extrapolation of the energies towards the complete basis set[21] were used to minimize the computational uncertainties of the relative conformational energies.

The computed energy using DLPNO-CCSD(T) method are quite close to those obtained from the CAM-B3LYP-D3 calculations. The differences between the energies obtained from the DFT and the *ab inito* methods do not exceed 11 meV (Table S10), which is less than a half of the thermal energy, k_BT, at room temperature. This finding suggests that the DFT estimates of the energies represent well the discussed trends and that ab initio energy refinement does not impart any qualitative perturbation.

Table S10. DFT and *ab initio* calculated energies (in eV) of the *E*-conformer and the two transition states, TS_1 and TS_2, relative to the energy of the low-lying *Z*-conformer.

solvent	CAM-B3LYP			DLPNO-CCSD(T)			difference		
	TS_1	*E*	TS_2	TS_1	*E*	TS_2	TS_1	*E*	TS_2
Vac	0.185	0.126	0.209	0.194	0.116	0.219	0.0091	-0.0098	0.0098
Tol	0.147	0.0944	0.169	0.148	0.0794	0.165	0.0012	-0.015	-0.0034
$CHCl_3$	0.122	0.0689	0.137	0.121	0.0561	0.134	-0.00039	-0.013	-0.0034
CH_2Cl_2	0.105	0.0506	0.115	0.110	0.0428	0.110	0.0052	-0.0078	-0.0043
CH_3CN	0.0864	0.0304	0.0904	0.0974	0.0270	0.0983	0.011	-0.0034	0.0079
DMSO	0.0858	0.0286	0.0890	0.0905	0.0255	0.0967	0.0047	-0.0031	0.0077
water	0.0827	0.0261	0.0859	0.0845	0.0240	0.0945	0.0017	-0.0021	0.0086

Implicit solvent models. To examine the validity of our selection of the widely-used polarizable continuum model (PCM) [22], in its integral equation formalism as the default implementation in Gaussian 16, we perform a comparative analysis of the shapes of the frontier orbitals of $\mathbf{Aaa^{(Z)}}$ conformer (Figure S14) and of the relative electronic energies and electric dipoles of $\mathbf{Aaa^{(Z)}}$ and $\mathbf{Aaa^{(E)}}$, and of the two transition states (Tables S11 and S12) using different models of implicit solvation: conductor-like polarizable continuum model (C-PCM) [23], self-consistent isodensity polarizable continuum model (SCIPCM) [24], solvation model based on the full density (SMD) [25], and Onsager reaction field with solute radius 5.52 Å [26]. All solvation models agree on the relative energies and dipole magnitudes of all conformers well within the chemical accuracy limits. Also, all solvation models captured quite similarly the trends of stabilization of $\mathbf{Aaa^{(E)}}$ and both transition states by solvation in polar media. The shapes of the frontier orbitals resulting from different solvation models are practically identical.

Table S11. Relative electronic energies (in kJ/mol) of the relevant **Aaa** conformers calculated at the CAM-B3LYP-D3(BJ)/6-311+G(d,p) level of theory implementing dichloromethane using various models of implicit solvation.

solvation model	*Z*	TS_1	*E*	TS_2
PCM	0.0	10.1	4.9	11.1
C-PCM	0.0	9.2	3.9	9.7
SCIPCM	0.0	11.0	4.8	12.2
SMD	0.0	10.6	4.6	11.9
Onsager	0.0	14.6	2.8	15.3

Table S12. Magnitudes of the electric dipole moments (in D) of the relevant Aaa conformers calculated at the CAM-B3LYP-D3(BJ)/6-311+G(d,p) level of theory and the CHELPG fitting procedure implementing dichloromethane using various models of implicit solvation.

solvation model	*Z*	TS_1	*E*	TS_2
PCM	4.618	7.414	10.626	8.505
C-PCM	4.660	7.569	10.823	8.707
SCIPCM	4.605	7.214	10.371	8.294
SMD	4.798	7.694	11.083	8.779
Onsager	4.600	6.654	9.608	7.549

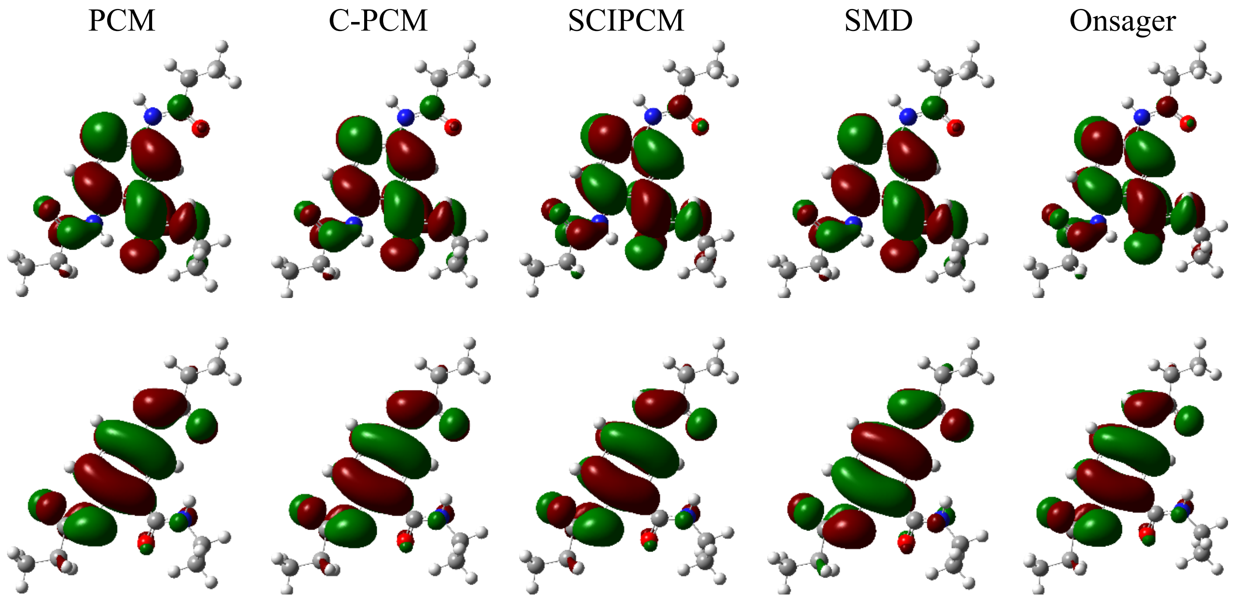

Figure S14. Comparison of the frontier molecular orbitals of **Aaa$^{(Z)}$** monomer calculated at the CAM-B3LYP-D3(BJ)/6-311+G(d,p) level of theory with implicit solvent, i.e., dichloromethane, introduced using various implicit solvation models. LUMO is depicted on the top row and HOMO is depicted on the bottom row.

SM References:

[1] E.M. Espinoza, J.M. Larsen-Clinton, M. Krzeszewski, N. Darabedian, D.T. Gryko, V.I. Vullev, Bioinspired approach toward molecular electrets: synthetic proteome for materials, Pure Appl. Chem., 89 (2017) 1777-1797.

[2] M. Krzeszewski, E.M. Espinoza, C. Cervinka, J.B. Derr, J.A. Clark, D. Borchardt, G.J.O. Beran, D.T. Gryko, V.I. Vullev, Dipole Effects on Electron Transfer are Enormous, Angew. Chem., Int. Ed., 57 (2018) 12365-12369.

[3] J.M. Larsen, E.M. Espinoza, J.D. Hartman, C.-K. Lin, M. Wurch, P. Maheshwari, R.K. Kaushal, M.J. Marsella, G.J. Beran, V.I. Vullev, Building blocks for bioinspired electrets: molecular-level approach to materials for energy and electronics, Pure Appl. Chem., 87 (2015) 779-792.

[4] J.M. Larsen-Clinton, E.M. Espinoza, M.F. Mayther, J. Clark, C. Tao, D. Bao, C.M. Larino, M. Wurch, S. Lara, V.I. Vullev, Fluorinated aminoanthranilamides: Non-native amino acids for bringing proteomic approaches to charge-transfer systems, PCCP, 19 (2017) 7871-7876.

[5] T. Shida, Electronic absorption spectra of radical ions, Elsevier Publishing Company, 1988.

[6] K. Bobrowski, Radiation-Induced Radical Reactions, in: Encyclopedia of Radicals in Chemistry, Biology and Materials.

[7] G. Jones, V.I. Vullev, Ground-and excited-state aggregation properties of a pyrene derivative in aqueous media, J. Phys. Chem. A, 105 (2001) 6402-6406.

[8] M.Y. Yang, O. O'Mari, W.A. Goddard III, V.I. Vullev, How Permanent Are the Permanent Macrodipoles of Anthranilamide Bioinspired Molecular Electrets?, JACS, 146 (2024) 5162-5172.

[9] T.O. Lopes, D.F.S. Machado, C. Risko, J.-L. Brédas, H.C. de Oliveira, Bond ellipticity alternation: an accurate descriptor of the nonlinear optical properties of π-conjugated chromophores, J. Phys. Chem. Lett., 9 (2018) 1377-1383.

[10] S. Emamian, T. Lu, H. Kruse, H. Emamian, Exploring nature and predicting strength of hydrogen bonds: a correlation analysis between atoms-in-molecules descriptors, binding energies, and energy components of symmetry-adapted perturbation theory, J. Comput. Chem., 40 (2019) 2868-2881.

[11] E.R. Johnson, S. Keinan, P. Mori-Sánchez, J. Contreras-García, A.J. Cohen, W. Yang, Revealing noncovalent interactions, JACS, 132 (2010) 6498-6506.

[12] T. Lu, F. Chen, Multiwfn: A multifunctional wavefunction analyzer, J. Comput. Chem., 33 (2012) 580-592.

[13] A.E. Reed, R.B. Weinstock, F. Weinhold, Natural population analysis, J. Chem. Phys., 83 (1985) 735-746.

[14] A.E. Reed, L.A. Curtiss, F. Weinhold, Intermolecular interactions from a natural bond orbital, donor-acceptor viewpoint, Chem. Rev., 88 (1988) 899-926.

[15] K. Wolinski, J.F. Hinton, P. Pulay, Efficient implementation of the gauge-independent atomic orbital method for NMR chemical shift calculations, JACS, 112 (1990) 8251-8260.

[16] K.R. Bryenton, A.A. Adeleke, S.G. Dale, E.R. Johnson, Delocalization error: The greatest outstanding challenge in density-functional theory, WIREs Computational Molecular Science, 13 (2023) e1631.

[17] C. Riplinger, P. Pinski, U. Becker, E.F. Valeev, F. Neese, Sparse maps—A systematic infrastructure for reduced-scaling electronic structure methods. II. Linear scaling domain based pair natural orbital coupled cluster theory, J. Chem. Phys., 144 (2016).

[18] G.L. Stoychev, A.A. Auer, F. Neese, Automatic generation of auxiliary basis sets, J. Chem. Theory Comput., 13 (2017) 554-562.

[19] F. Neese, F. Wennmohs, U. Becker, C. Riplinger, The ORCA quantum chemistry program package, J. Chem. Phys., 152 (2020).

[20] Y. Guo, C. Riplinger, U. Becker, D.G. Liakos, Y. Minenkov, L. Cavallo, F. Neese, Communication: An improved linear scaling perturbative triples correction for the domain based local pair-natural orbital based singles and doubles coupled cluster method [DLPNO-CCSD (T)], J. Chem. Phys., 148 (2018).

[21] F. Neese, E.F. Valeev, Revisiting the atomic natural orbital approach for basis sets: robust systematic basis sets for explicitly correlated and conventional correlated ab initio methods?, J. Chem. Theory Comput., 7 (2011) 33-43.

[22] G. Scalmani, M.J. Frisch, Continuous surface charge polarizable continuum models of solvation. I. General formalism, J. Chem. Phys., 132 (2010) 114110.

[23] M. Cossi, N. Rega, G. Scalmani, V. Barone, Energies, structures, and electronic properties of molecules in solution with the C-PCM solvation model, Journal of Computational Chemistry, 24 (2003) 669-681.

[24] J.B. Foresman, T.A. Keith, K.B. Wiberg, J. Snoonian, M.J. Frisch, Solvent Effects. 5. Influence of Cavity Shape, Truncation of Electrostatics, and Electron Correlation on ab Initio Reaction Field Calculations, J. Phys. Chem., 100 (1996) 16098-16104.

[25] A.V. Marenich, C.J. Cramer, D.G. Truhlar, Universal Solvation Model Based on Solute Electron Density and on a Continuum Model of the Solvent Defined by the Bulk Dielectric Constant and Atomic Surface Tensions, J. Phys. Chem. B, 113 (2009) 6378-6396.

[26] M.W. Wong, K.B. Wiberg, M. Frisch, Hartree–Fock second derivatives and electric field properties in a solvent reaction field: Theory and application, J. Chem. Phys., 95 (1991) 8991-8998.
